# Interaction of the Ankyrin H Core Effector of *Legionella* with the Host LARP7 Component of the 7SK snRNP Complex

**DOI:** 10.1128/mBio.01942-19

**Published:** 2019-08-27

**Authors:** Juanita Von Dwingelo, Ivy Yeuk Wah Chung, Christopher T. Price, Lei Li, Snake Jones, Miroslaw Cygler, Yousef Abu Kwaik

**Affiliations:** a Department of Microbiology and Immunology, University of Louisville, Louisville, Kentucky, USA; b Department of Biochemistry, University of Saskatchewan, Saskatoon, Saskatchewan, Canada; c Department of Biochemistry, McGill University, Montreal, Quebec, Canada; d Center for Predictive Medicine, College of Medicine, University of Louisville, Louisville, Kentucky, USA; University of Nebraska Medical Center; Technische Universität Braunschweig; Northwestern University Medical School; Ohio State University; Tufts Medical School

**Keywords:** AnkH, Dot/Icm type IVB secretion system, LARP7, *Legionella pneumophila*, effector functions, transcriptional regulation

## Abstract

Species of the *Legionella* genus encode at least 18,000 effector proteins that are translocated through the Dot/Icm type IVB translocation system into macrophages and protist hosts to enable intracellular growth. Eight effectors, including ankyrin H (AnkH), are common to all *Legionella* species. The AnkH effector is also present in *Coxiella* and *Rickettsiella*. To date, no pathogenic effectors have ever been described that directly interfere with host cell transcription. We determined that the host nuclear protein La-related protein 7 (LARP7), which is a component of the 7SK small nuclear ribonucleoprotein (snRNP) complex, interacts with AnkH in the host cell nucleus. The AnkH-LARP7 interaction partially impedes interactions of the 7SK snRNP components with LARP7, interfering with transcriptional elongation by polymerase (Pol) II. Consistent with that, our data show AnkH-dependent global reprogramming of transcription of macrophages infected by Legionella pneumophila. The crystal structure of AnkH shows that it contains four N-terminal ankyrin repeats, followed by a cysteine protease-like domain and an α-helical C-terminal domain. A substitution within the β-hairpin loop of the third ankyrin repeat results in diminishment of LARP7-AnkH interactions and phenocopies the *ankH* null mutant defect in intracellular growth. LARP7 knockdown partially suppresses intracellular proliferation of wild-type (WT) bacteria and increases the severity of the defect of the Δ*ankH* mutant, indicating a role for LARP7 in permissiveness of host cells to intracellular bacterial infection. We conclude that the AnkH-LARP7 interaction impedes interaction of LARP7 with 7SK snRNP, which would block transcriptional elongation by Pol II, leading to host global transcriptional reprogramming and permissiveness to L. pneumophila.

## Abstract (precis)

For intracellular pathogens to thrive in host cells, an environment that supports survival and replication needs to be established. L. pneumophila accomplishes this through the activity of the ∼330 effector proteins that are injected into host cells during infection. Effector functions range from hijacking host trafficking pathways to altering host cell machinery, resulting in altered cell biology and innate immunity. One such pathway is the host protein synthesis pathway. Five L. pneumophila effectors have been identified that alter host cell translation, and 2 effectors have been identified that indirectly affect host cell transcription. No pathogenic effectors have been described that directly interfere with host cell transcription. Here we show a direct interaction of the AnkH effector with a host cell transcription complex involved in transcriptional elongation. We identify a novel process by which AnkH interferes with host transcriptional elongation through interference with formation of a functional complex and show that this interference is required for pathogen proliferation.

## INTRODUCTION

Legionella pneumophila is a Gram-negative intracellular pathogen that is ubiquitous in freshwater environments (1), where it primarily parasitizes a wide range of protozoan hosts, which serve as the bacterial natural hosts (2–5) and contribute to the pathogenesis and ecology of the pathogen (6–10). When humans encounter contaminated water sources, aerosolized water droplets can be inhaled and reach the lung, where bacteria can invade and proliferate within alveolar macrophages, causing pneumonia (1, 10). To date, approximately 65 species of *Legionella* have been identified, with almost half of the species having been found to be capable of causing disease in humans (11–14). L. pneumophila in particular is responsible for 90% of Legionnaires’ disease cases globally (15).

The life cycles of L. pneumophila within amoebae and within alveolar macrophages are strikingly similar (5, 16–20). After the bacteria are engulfed by the cell, endoplasmic reticulum (ER)-derived vesicles fuse to the phagosome to generate the *Legionella*-containing vacuole (LCV) (18–23), which evades the host endosomal-lysosomal degradation pathway but communicates with early secretory vesicle trafficking pathways (24, 25). Biogenesis of the LCV is dependent on the Dot/Icm type IV secretion system (T4SS), which is responsible for translocation of at least 330 effector proteins into the host cell cytoplasm (26, 27). The injected effectors interact with specific host targets to modulate a plethora of host cell processes that remodel the macrophage and amoeba host into a proliferative niche (26, 28–30). In most cases, the deletion of a single L. pneumophila effector gene does not result in a growth defect in mammalian macrophages or amoeba (31). Although this is thought to be due to redundancy, it is more likely that many of the effectors in this arsenal are host specific and constitute a “toolbox” and that specific tools are utilized in specific environmental eukaryotic hosts (24, 32). Genomic analyses of 58 *Legionella* species have shown that the *Legionella* genus has ∼18,000 effectors but that only 8 of these effectors (MavN, VipF, RavC, CetLp1, lpg2832, lpg3000, lpg1356/lpp1310, and AnkH/LegA3/Lpg2300) are conserved among all *Legionella* species and are designated core effectors (12, 14). Among the 8 core effectors, AnkH is the only one that is conserved among all bacterial pathogens harboring the Dot/Icm T4SS, including Coxiella burnetii and Rickettsiella grylli (12, 14). It is therefore likely that AnkH is involved in altering an evolutionarily conserved eukaryotic process required for the infection by many obligate and facultative intracellular pathogens.

A large number of the Dot/Icm-translocated effector proteins contain eukaryote-like motifs and domains, which is likely the result of long-term coevolution of L. pneumophila with its various protozoan hosts, leading to interkingdom horizontal gene transfer (9, 32–37). Examples of these eukaryotic domains include F box and prenylation motifs, the U box domain, leucine-rich repeats, and ankyrin repeat domains (ARDs), which are protein-protein interaction domains (38–42).

The ankyrin repeat (AR) is a structural fold composed of two α-helices forming a helix-turn-helix motif. It is one of the most commonly found structural motifs in eukaryotic proteins (34, 38). Each AR-containing domain (ARD) usually contains multiple ARs (43–48), functioning predominantly as protein-protein interaction scaffolds (49, 50). Many bacterial pathogens that inject protein effectors into host cells harbor eukaryote-like ARD-containing protein effectors that interact with specific host targets (33, 51, 52). Among 58 sequenced species of *Legionella*, 1,134 ARD-containing effectors have been identified in various combinations with other eukaryotic domains (12, 14, 53).

While many L. pneumophila effectors are dispensable for intracellular growth of the pathogen in macrophages, we have previously shown that the AnkH ARD-containing effector is one of very few effectors required for intracellular replication in macrophages and amoebae and for intrapulmonary proliferation in the A/J mouse model (53, 54). We have also shown that AnkH is among the effector proteins that contain an asparagine hydroxylation motif [Lxxxxx(D/E)(ILVA)N(ILVA)], which is hydroxylated in human macrophages (54, 55).

While no L. pneumophila effectors have been shown to interfere directly with host transcription machinery, few L. pneumophila effectors have been identified that modulate host translation machinery. Five effectors (Lgt1, Lgt2, Lgt3, SidI, and SidL) act on host translation machinery primarily by interfering with the eELF1A and eELF1Bγ host elongation factors (56–59). In contrast, the RomA (or LegAS4) effectors are SET domain-containing proteins that directly modify host chromatin through histone modification; however, the effect on host transcription is not known (60, 61). The LegK7 effector interferes with the host Hippo signaling pathway, which results in the degradation of TAZ and YAP1 transcriptional regulators, altering the transcriptional profile of mammalian macrophages (62).

No bacterial effector has been shown to modulate the function of 7SK small nuclear ribonucleoprotein (7SK snRNP). La-related protein 7 (LARP7) is a component of the 7SK snRNP complex which controls the pausing time of polymerase (Pol) II at the initiation of transcriptional elongation at almost all metazoan genes (63–65). Binding of LARP7 to the 7SK 3′-terminal U-rich sequence protects 7SK from nucleolytic degradation (65–69). The canonical 7SK snRNP core complex consists of 7SK, LARP7, and γ-methylphosphate capping enzyme (MePCE) (63–65). Formation of the 7SK snRNP core complex enables recruitment of transcription elongation factor b (P-TEFb; Cdk9-cyclin T1 heterodimer) and HEXIM1/2 dimer to the complex (64, 67, 70–73). Binding and sequestration of P-TEFb within the 7SK snRNP complex result in inhibition of its kinase activity and continuation of the pause in Pol II transcription elongation (66, 69, 74, 75). P-TEFb is the critical factor that controls the release of paused Pol II into productive elongation at almost all metazoan genes. Various stimuli trigger the release of P-TEFb from the 7SK snRNP complex, leading to activation of its kinase activity and transition of Pol II into productive transcriptional elongation (76, 77). Our data indicate that the β-hairpin loop of the third ankyrin repeat of AnkH interacts with LARP7. The AnkH-LARP7 interaction impedes interaction of LARP7 with the 7SK snRNP complex components, which would trigger transcriptional elongation by Pol II leading to host global transcriptional reprogramming.

## RESULTS

### Interaction of AnkH with the LARP7 host protein.

We utilized the yeast two-hybrid (Y2H) system to identify potential host cell interacting partners of AnkH. The full-length coding sequence of AnkH served as the bait construct, and the normalized universal human library was used for the prey. After mating of the two yeast strains, a total of 1,004 potentially positive clones were identified, and their growth on a selective media narrowed the number of clones representing positive results to 37. After multiple rounds of cotransformations of AnkH and the 37 positive clones, 7 potential interacting partners of AnkH were identified (Table 1). Of the seven host protein candidates, LARP7 was the only one testing positive in all cotransformations; thus, we pursued verification of its interaction with AnkH.

**TABLE 1:** Potential interacting partners identified in Y2H screen

Protein identified by yeast two-hybrid assay	Protein function
LA-related protein 7 (LARP7)	Involved in global transcription regulation
Intersectin 2 (INST2)	Adaptor protein involved in trafficking of endocytic vesicles
Ubiquitin-specific peptidase-like 1 (USPL1)	SUMO-specific isopeptidase involved in protein deSUMOylation
ANK repeat domain 18A (ANKRD18A)	Possible role in global regulation of platelet function and no.
TOX4	Involved in regulating chromatin structure and cell cycle progression
Sodium channel modifier 1 (SNCM1)	Zinc finger protein and putative splicing factor
HLA-DQA1	Involved in process of presenting antigens on cell surface

**FIG 1:**
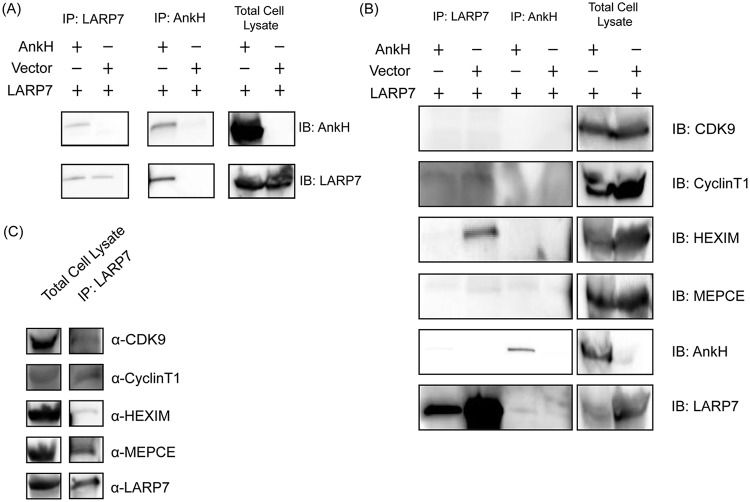
Interaction of LARP7 with the AnkH effector. (A) HEK293T cells were transiently transfected with 3XFLAG-AnkH or 3XFLAG-BAP and c-*myc*-LARP7 and immunoprecipitated with anti-FLAG or anti-myc antibody, and the co-IP was subjected to immunoblotting to detect the presence of AnkH and LARP7. (B) The AnkH co-IP was subjected to immunoblotting (IB) against 7SK snRNP complex components. (C) HEK293T cells were transiently transfected with c-*myc*-LARP7 and immunoprecipitated with anti-myc antibody, and the IP was subjected to immunoblotting to detect the presence of 7SK snRNP complex components. Lanes for total cell lysates of the immunoblot were imaged for less time due to a high-intensity signal. Results are representative of five independent experiments.

The LARP7 protein is a component of the 7SK snRNP complex, which enables a continued pause of Pol II elongation through sequestering and inhibiting the kinase activity of P-TEFb (78). To confirm the AnkH-LARP7 interaction, tagged AnkH and LARP7 were cotransfected into human embryonic kidney (HEK293T) cells and subjected to reciprocal coimmunoprecipitation (co-IP) by IP of AnkH or LARP7 (Fig. 1A). The data showed that LARP7 was pulled down with AnkH, in the reciprocal co-IPs (Fig. 1A). To determine if AnkH-LARP7 interaction impacted recruitment of critical components essential for sequestration of P-TEFb in the 7SK snRNP complex, we determined whether the LARP7-AnkH complex interacted with the 7SK snRNP components. The AnkH co-IP was probed using immunoblotting for components of the 7SK snRNP complex (CDK9, cyclin T1, MePCE, HEXIM1/2). The data showed that none of the other complex components were immunoprecipitated with the LARP7-AnkH complex, similarly to the vector control (Fig. 1B). However, MePCE was immunoprecipitated with the LARP7-AnkH complex 60% of the time (3 of 5 replicates). This could have been the result of expression of MePCE and of the transient formation of the 7SK snRNP complex or could have indicated that these MePCE-positive samples were immunoprecipitated in instances where LARP7 was part of the complex and had not yet been removed from the complex via the LARP7-AnkH interaction. Importantly, in the absence of AnkH, all of the 7SK snRNP components immunoprecipitated in a complex with LARP7 (Fig. 1C). Our data show that AnkH specifically interacts *in vivo* with the LARP7 protein and that this impedes interaction of LARP7 with critical components of the 7SK snRNP complex required for the sequestration of P-TEFb in the 7SK snRNP complex.

### Localization of AnkH with LARP7 to the host cell nucleus.

**FIG 2:**
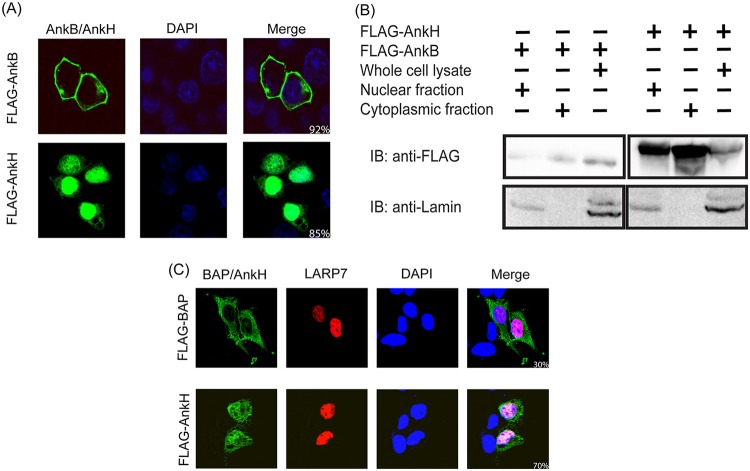
Localization of AnkH with LARP7 in the nucleus. (A) Representative confocal microscopy images of HEK293T cells transiently transfected with 3XFLAG-AnkH or 3XFLAG-AnkB control. The cells were labeled with anti-FLAG antibody (green), and the nucleus was stained with DAPI (blue). (B) HEK293T cells were transiently transfected with 3XFLAG-AnkH or a 3XFLAG-AnkB control and were subjected to nuclear fractionation. Cell fractions were separated by SDS-PAGE and analyzed by immunoblotting. AnkH and AnkB were detected using anti-FLAG monoclonal antibody. Fractionation was confirmed by detection of the nuclear protein lamin. (C) Representative confocal microscopy images of HEK293T cells transiently cotransfected with 3XFLAG-AnkH and c-*myc*-LARP7 or 3XFLAG-BAP and c-*myc*-LARP7. The cells were labeled with anti-FLAG (green) or anti-myc (red), and the nucleus was stained with DAPI (blue). Numbers in the merged images in panels A and C represent results of quantification of percentages of nuclear localizations of AnkH and LARP7 proteins in HEK293T cells. For panels A and C, 100 transfected cells were analyzed from multiple coverslips. Results are representative of three independent experiments performed in triplicate.

Consistent with its role in transcription, LARP7 is localized primarily in the nucleus (78). Since AnkH interacts with LARP7, we determined whether the AnkH effector was targeted to the nucleus. HEK293T cells were transfected with a plasmid containing tagged AnkH, and subcellular localization of AnkH was examined using confocal microscopy (Fig. 2A). In 85% of transfected cells, the AnkH effector was predominantly localized to the nucleus in addition to showing some cytosolic localization (Fig. 2A). In contrast, the AnkB effector control was primarily (92%) localized to the plasma membrane (Fig. 2A) (40, 79).

To confirm subcellular localization of AnkH, nuclear and cytoplasmic fractions were analyzed by immunoblotting. In cells transfected with tagged AnkH, similar amounts of AnkH were present in the nuclear and cytoplasmic fractions (Fig. 2B), while the AnkB effector control was mainly localized to the cytoplasmic fraction (Fig. 2B). Cellular fractionation was confirmed using the nuclear protein lamin as a control (Fig. 2B).

To determine if AnkH and LARP7 were simultaneously localized to the nucleus, HEK293T cells were transfected with tagged AnkH and LARP7 and confocal microscopy was performed. The tagged bacterial alkaline phosphatase (BAP) was used as the control. Our data confirmed that ∼70% of the cells showed simultaneous localization of AnkH and LARP7 in the nucleus (Student's *t* test; *P < *0.01) (Fig. 2C) compared to the proportion (∼30%) seen with the BAP control, which is a highly expressed protein (Fig. 2C). Our data showed that AnkH and LARP7 are localized to the nucleus, consistent with their interaction.

### Role of LARP7 in intracellular replication of L. pneumophila in hMDMs.

**FIG 3:**
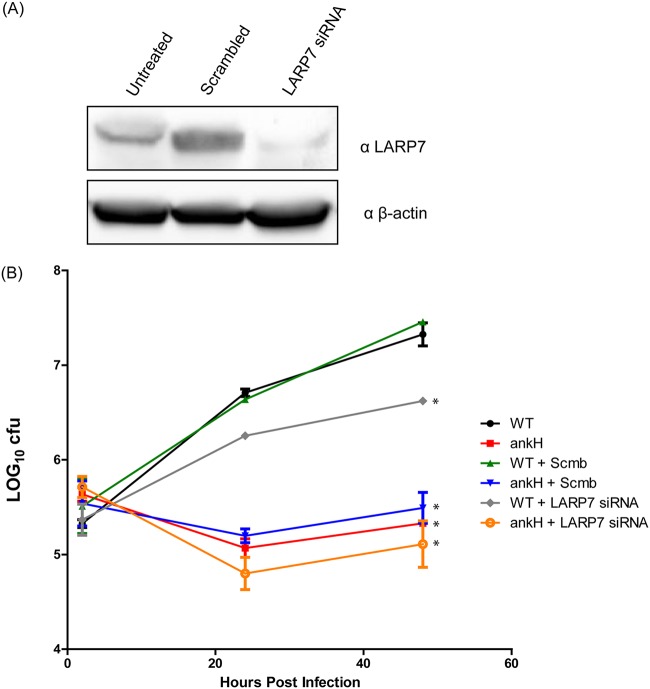
Requirement of LARP7 for intracellular replication of L. pneumophila. (A) Cells were treated with LARP7 RNAi for 24 h then infected. Knockdown of LARP7 was determined by immunoblotting with anti-LARP7 polyclonal antibody. (B) Intracellular growth kinetics of L. pneumophila in hMDMs treated with LARP7-specific or scrambled RNAi. The results are representative of three independent experiments performed in triplicate. Statistical analysis was performed using Student's *t* test (*, *P < *0.05).

We have previously shown that AnkH is required for intracellular replication of L. pneumophila in macrophage and amoeba (53, 54). Depletion of either LARP7 or MePCE via RNA interference (RNAi) triggers 7SK degradation in cells (67, 71, 80). Since AnkH interacts with the LARP7 component of the 7SK snRNP complex, we investigated if LARP7 was also required for replication of L. pneumophila. We utilized a lentiviral RNAi system to knockdown expression of LARP7 in human monocyte-derived macrophages (hMDMs) that were infected with the wild-type (WT) strain of L. pneumophila or with the Δ*ankH* null mutant. Knockdown of LARP7 was confirmed by immunoblotting (Fig. 3A). Interestingly, when LARP7 was knocked down and cells were infected with the Δ*ankH* mutant, the defective phenotype was exacerbated. Surprisingly, the knockdown of LARP7 resulted in a partial but significant decrease in intracellular replication of the WT strain (Student's *t* test; *P < *0.05) which was not observed in nontreated or control RNAi-treated cells (Fig. 3B). These data support our findings with respect to the role of AnkH-LARP7 interaction in intracellular replication of L. pneumophila in hMDMs and indicate that LARP7 is involved in transcription of genes involved in permissiveness to L. pneumophila.

### Alteration of host global transcription by AnkH.

Our data showed that the LARP7-AnkH complex impedes interactions of LARP7 with critical components of the 7SK snRNP complex required for the sequestration of P-TEFb in the 7SK snRNP complex, which indicates the presence of an active P-TEFb kinase and release of Pol II from pause sites, and transitions into productive transcriptional elongation (67, 78). We utilized transcriptome sequencing (RNA-Seq) to examine modulation of global gene expression in hMDMs infected with either the WT strain or the Δ*ankH* null mutant. The data showed that AnkH had a dramatic effect on global transcription of L. pneumophila-infected hMDMs, with a total of 405 genes that were differentially regulated in cells infected with the WT strain compared to the Δ*ankH* mutant; the top 10 of each (based on log fold change) are listed in Table 2 (a full list is provided in Table S3 in the supplemental material [see also Table S4]). MetaCore was used to determine which pathways were differentially regulated based on *P* values. Certain cellular pathways, including the apoptosis pathway and the autophagy pathway, and certain signaling pathways, including the STK3/4 pathway and Jun N-terminal protein kinase (JNK) pathway, were downregulated in an AnkH-dependent manner, indicating negative regulation of these pathways by AnkH during infection (Table 3). Transcription and immune response pathways were downregulated in cells infected with the *ΔankH* null mutant compared to cells infected with the WT strain, indicating their upregulation by AnkH (Table 3). These data show that AnkH triggers a dramatic reprogramming of cellular transcription and that the reprogramming is most likely mediated by interaction with several host substrates, one of which is LARP7.

**TABLE 2:** Top 10 upregulated genes and top 10 downregulated genes in cells infected with *ankH* mutant compared to cells infected with WT

Gene symbol (description)	Log_2_ FC^*a*^
HSPA1B (heat shock 70-kDa protein 1B)	+2.9979
EGR1 (early growth response 1)	+2.25815
DNAJB1 (DnaJ [Hsp40] homolog, subunit B, member 1)	+1.51215
DUSP1 (dual-specificity phosphatase 1)	+1.24951
FOS (FBJ [Finkel-Biskis-Jinkins] murine osteosarcoma viral oncogene homolog)	+1.15258
TDO2 (tryptophan metabolism)	+1.01074
MS4A4E (membrane-spanning 4-domains, subfamily A, member 4E)	+2.36464
PKIB (protein kinase inhibitor beta)	+1.33129
PEG3 (paternally expressed 3)	+2.64637
GRIK2 (glutamate receptor, ionotropic, kainate 2)	+1.78851
TMC8 (transmembrane channel-like 8)	−2.75204
HMHA1 (histocompatibility [minor] HA-1)	−1.99385
DAPK3 (death-associated protein kinase 3)	−1.91505
PDLIM2 (PDZ and LIM domain 2)	−1.79007
SDF2L1 (stromal cell-derived factor 2-like 1)	−1.65596
TOR2A (torsin family 2, member A)	−1.62058
LMF2 (lipase maturation factor 2)	−1.59493
NOTCH3 (Notch 3)	−1.57732
IL-27 (interleukin 27)	−1.55472
CPSF1 (cleavage and polyadenylation-specific factor 1)	−1.89935
TMC8 (transmembrane channel-like 8)	−2.75204

a Log_2_ FC, log_2_ fold change.

**TABLE 3:** Upregulated and downregulated pathways in cells infected with *ankH*^*a*^

Upregulated pathway	*P* value	Downregulated pathway	*P* value
Development-positive regulation of STK3/4 (Hippo) pathway and negative regulation of YAP/TAZ function	1.338e−9	Transcription, HIF-1 targets	2.822e−15
Transport clathrin-coated vesicle cycle	2.291e−9	Immune response, IL-3 signaling via JAK/STAT, p38, JNK, and NFκB	1.745e−14
Apoptosis and survival, FAS signaling cascades	9.334e−9	Immune response, IL-1 signaling pathway	1.270e−11
Immune response, antigen presentation by MHC class I (cross-presentation)	5.959e−8	Immune response, IL-10 signaling pathway	1.397e−11
Signal transduction, JNK pathway	6.740e−8	Apoptosis and survival, anti-apoptotic TNF/NFκB/Bcl-2 pathway	2.725e−11

a FAS, fatty acid synthase; JNK, Jun N-terminal protein kinase; IL-3, interleukin-3; MHC, major histocompatibility complex; TNF, tumor necrosis factor.

10.1128/mBio.01942-19.3 TABLE S3 Complete list of genes upregulated in hMDMs infected with the Δ*ankH* null mutant compared to the WT strain of L. pneumophila. Download Table S3, DOCX file, 0.02 MB. Copyright © 2019 Von Dwingelo et al. 2019 Von Dwingelo et al. This content is distributed under the terms of the Creative Commons Attribution 4.0 International license.

10.1128/mBio.01942-19.4 TABLE S4 Complete list of genes downregulated in hMDMs infected with the Δ*ankH* null mutant compared to WT strain of L. pneumophila. Download Table S4, DOCX file, 0.1 MB. Copyright © 2019 Von Dwingelo et al. 2019 Von Dwingelo et al. This content is distributed under the terms of the Creative Commons Attribution 4.0 International license.

### The crystal structure of AnkH.

**FIG 4:**
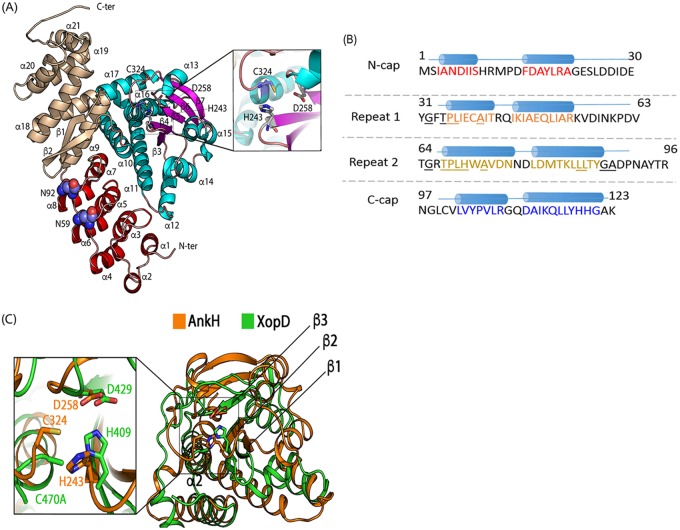
The crystal structure of AnkH. (A) AnkH consists of 3 domains: N-terminal ankyrin domain (α1 to α8; red), the cysteine proteinase-like domain (α10 to α17 and β3 to β7; cyan and magenta), and the cap domain (β1 and β2, α9, and α18 to α21; wheat). The inset shows a closeup of putative catalytic triad residues H243, D258, and C324. The HIF hydroxylation sites (N59 and N92) are located within the N-terminal domain and are shown in a sphere representation (blue and red). (B) Primary sequence of ankyrin domain. The length of each ankyrin repeat was determined using the consensus sequence based on statistical analysis of 4,000 ankyrin repeat sequences from the PFAM database as proposed by Mosavi et al. (47). Highlighted (colored) letters correspond to α-helices for each domain. The conserved residues are underlined, and the a-helices are shown as cylinders. (C) Superposition of AnkH with *Xanthomonas* XopD C470A mutant. The cartoon diagram represents superposition of the AnkH cysteine protease-like domain (residues 163 to 342; orange) and the *Xanthomonas* XopD C470A mutant (PBD identifier ID 2OIX, residues 336 to 515; green). The three β-strands and two α-helices that form the core of the domains and overlap well are marked. The inset shows a closeup of the catalytic triad. In AnkH, these residues are His243, Asp258, and Cys324; in XopD, these residues are His409, Asp429, and Cys470.

AnkH is one of a few of the ∼330 *Legionella* effectors required for intracellular growth within amoebal hosts and human macrophages (9, 32). To gain greater insight into possible cellular functions of AnkH, we determined its three-dimensional crystal structure. AnkH is an α/β-fold protein and contains a total of 21 α-helices and seven β-strands (Fig. 4A). AnkH consists of 3 domains: N-terminal ankyrin domain (α1 to α8; see red data in Fig. 4A), the middle domain (α10 to α17 and β3 to β7; cyan and magenta), and the cap domain (β1 to β2, α9, and α18 to α21; wheat (53, 54). The N-terminal domain contains ankyrin repeats with four helix-turn-helix repeats (α1 to α8, residues 1 to 122) (Fig. 4B). The first repeat is somewhat distorted and has shorter α-helices. The ARD is followed by a 4-turn-long helix α9 and an extended β-hairpin (β1 to β2, residues 123 to 162) leading to the middle domain (Fig. 4A). This domain (residues 163 to 361) contains a central 5-stranded antiparallel β-sheet, β3 to β7, and is extended by the presence of helix α12. The β-sheet is flanked by two layers of two helices (inner, α11 and α16; outer, α10 and α17) on one side and two helices (α14 and α15) on the other side. The C-terminal domain (residues 362 to 461) contains a five-helix bundle (Fig. 4A) and packs tightly together with α9 and the following β-hairpin, forming one domain. The N-terminal and C-terminal domains pack end to end into a crescent shape (Fig. 4A). The middle domain forms an independent insertion attached to the side of the ARD that typically functions as the protein binding surface. The long loops emanating from the ARD, usually involved in protein-protein interactions, face the middle domain.

### The inserted middle domain of AnkH has a cysteine protease fold.

To gain insight into possible functions of the middle and cap domains, we searched for their structural homologs using the Dali server (81). The middle (insertion) domain showed structural similarity to several proteins with a cysteine protease fold, albeit with relatively low scores. This cysteine protease-like domain (CPLD) is most similar to the outer protein D (XopD; PDB identifier [ID] 2OIX) from bacterial plant pathogen Xanthomonas campestris pv. *vesicatoria* (82–84) (Fig. 4C). It also shows similarity to a domain of another Legionella pneumophila effector, RavZ (85, 86).

XopD belongs to the ubiquitin-like-specific protease 1 family (87) and is classified within clan CE in the MEROPS database (88), with the catalytic triad arranged in the following order: histidine, glutamate/aspartate/asparagine, and cysteine. Cysteine functions as a nucleophile, while histidine serves as a general base and is in turn stabilized by glutamic acid/aspartic acid (87). The structure-based sequence identity between the aligned regions of CPLD and XopD is only ∼12%; nevertheless, three β-strands and two α-helices are structurally similar between AnkH and XopD (Fig. 4C), with His243, Asp258, and Cys324 of AnkH superposed on the catalytic triad of XopD. The histidine resides on the N-terminal end of the conserved strand within the protease fold (β4 in AnkH; Fig. 4A). The stabilizing aspartic acid sits at the C-terminal end of the conserved antiparallel strand (β5 in AnkH; Fig. 4A). The cysteine nucleophile is at the end of a long loop leading to the penultimate helix of the protease fold (Fig. 4A). The orientation of these three side chains in AnkH deviates from the active configuration, and a small rearrangement of the triad side chains has to occur to attain the active state (Fig. 4C). The fold of AnkH CPLD was recognized due to very low sequence identity to other cysteine protease and is not yet classified in the peptidase database MEROPS (89), which already includes several other peptidases from the *Legionella* species (data not shown).

### Structure-function analysis of AnkH.

**FIG 5:**
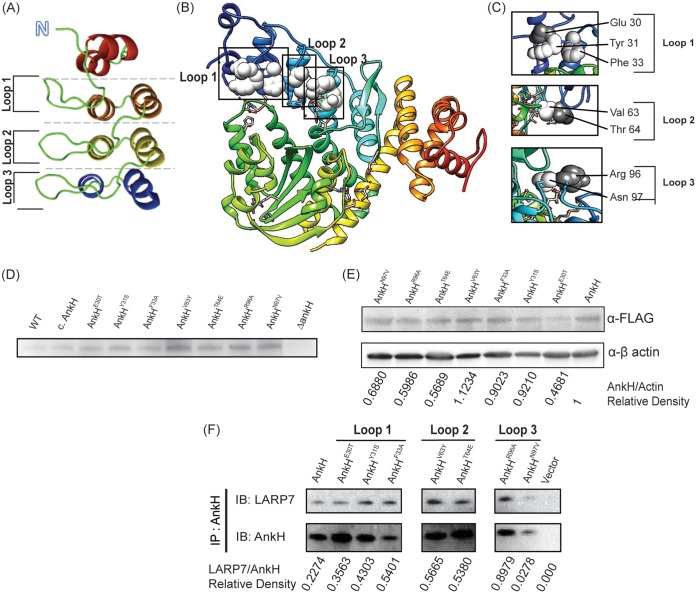
Substitutions in ARDs alter binding efficiency of AnkH and LARP7. (A) The ankyrin domain of AnkH shown as a ribbon diagram. The ankyrin domain consists of four ankyrin repeats: N-cap, repeat 1, repeat2, and C-cap. (B and C) Crystal structure of AnkH illustrating the whole structure (B) and insets representing different locations within the ARDs where residues were substituted (C). (D) Bacterial lysates from WT L. pneumophila and each of the AnkH substitution mutant strains were tested by immunoblotting for AnkH to determine protein stability. Cell lysates were subjected to immunoblotting to detect the presence of AnkH using goat anti-AnkH (53, 56). Equal numbers of bacteria were lysed for each strain. (E) HEK293T cells were transiently transfected with 3XFLAG-AnkH or the indicated 3XFLAG-AnkH substitution mutants and c-*myc*-LARP7. Densitometry was determined in accordance as actin ratio. (F) Cell lysates were immunoprecipitated with anti-FLAG antibody, and the co-IP was subjected to immunoblotting to detect the presence of AnkH and LARP7. Densitometry of the blotswas determined as the LARP7-to-AnkH ratio.

The structure of AnkH suggested that it binds a cellular target(s) through the β-hairpin loops within ARD and has a predicted proteolytic activity (Fig. 4A). To better understand the roles of the AnkH domains and to validate its structure, a total of 12 residues were chosen for single substitutions based on their location within a specific domain (Table 4; see also Fig. 4). The substituted residues included residues on the extended β-hairpin loops of ARs (Fig. 1; see also Fig. 4B), the putative cysteine protease catalytic triad and two asparagine residues (N59 and N92) that have been reported to undergo asparagine hydroxylation, which impacts protein-protein interactions (55). Each of the ARDs is illustrated in Fig. 5A. Panels B and C of Fig. 5 illustrate the location of each substitution made within the ARDs. The mutations had no detectable effect on stability of the variant proteins in L. pneumophila (Fig. 5D) or during transient transfection (Fig. 5E).

**TABLE 4:** Point mutations generated in different domains of AnkH^*a*^

ANK1 mutation(s)	ANK2 mutation(s)	ANK3 mutation(s)	Asn hydroxide mutation(s)	Cysteine-protease mutation(s)
E30T	V63Y	R96A	N59A	H243D
Y31S	T64E	N97V	N92A	D258A
F33A				C324S

a ANK1, ANK2, and ANK3 designate ankyrin repeat 1, ankyrin repeat 2, and ankyrin repeat 3.

HEK293T cells were cotransfected with LARP7 and either native AnkH or AnkH containing substitutions within the β-hairpin loops of the ARDs and were then subjected to immunoprecipitation. Our data showed that substitutions of residues within the ARD3 (specifically, Asn97) diminished the LARP7-AnkH interaction (Fig. 5F). In contrast, substitution of residue 30, 31, 33, 63, 64, or 96 resulted in enhanced binding between LARP7 and AnkH (Fig. 5F).

**FIG 6:**
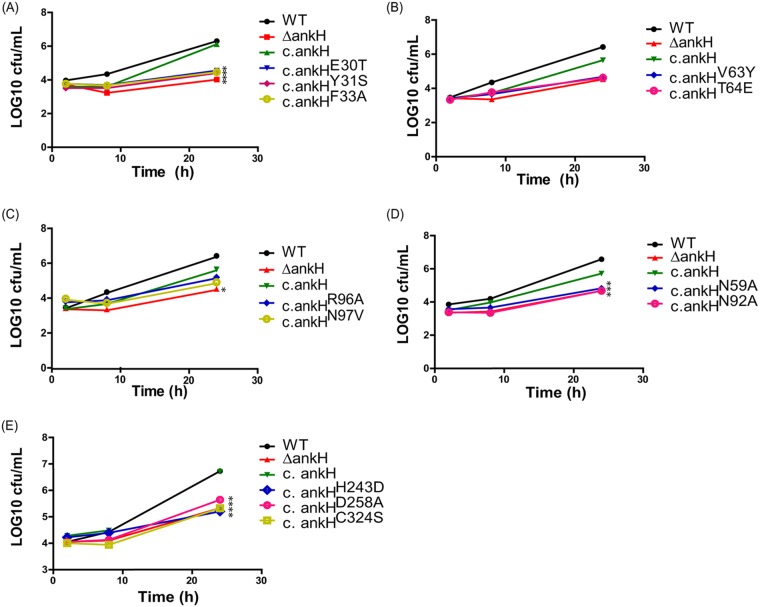
Structure-function analysis of AnkH in intracellular growth of L. pneumophila within hMDMs. Intracellular growth kinetics were determined for the WT strain, the *ankH* mutant, or the *ankH* mutant complemented with the WT allele (c.ankH) or with single and multiple substitution variants as indicated. All strains represented in all the panels were tested using the same WT control. (A) Mutations within first ANK repeat. (B) Mutations within second ANK repeat. (C) Mutations within third ANK repeat. (D) Mutations within the asparagine hydroxylation motif. (E) Mutations within the cystine-like protease pocket. The results are representative of three independent experiments performed in triplicate. Statistical analysis was performed using Student's *t* test (*, *P < *0.05).

In order to determine if the substitutions affected the function of AnkH in intracellular replication of L. pneumophila, hMDMs were infected with the WT strain, the Δ*ankH* null mutant, the Δ*ankH* mutant complemented with the WT allele of *ankh*, or the substitution variants of AnkH. Our data showed that substitution in the β-hairpin loop of ARD3, which led to reduced binding of LARP7 to AnkH, resulted in reduced intracellular growth of L. pneumophila (Fig. 6). All other residues selected for substitutions were partially required for AnkH function in intracellular replication, since introducing these mutations resulted in various degrees of partial replication defect compared to the WT strain (Student's *t* test; *P < *0.05) (Fig. 6) (Table 4). Therefore, we conclude that the ARD (in particular, Asn97), the cysteine-like protease domain, and the asparagine hydroxylation motifs are all required for the function of AnkH in intracellular proliferation of L. pneumophila within hMDMs.

## DISCUSSION

Among the ∼18,000 effectors of *Legionella* genus, AnkH is the only one conserved among all *Legionella* species as well as other pathogens that harbor the Dot/Icm secretion system (12, 14). While many L. pneumophila effectors are dispensable for intracellular growth of the pathogen in macrophages (24, 29, 32, 90), the AnkH effector plays a role in intracellular growth of L. pneumophila within amoeba hosts and within macrophages (53, 54). In addition, the high level of conservation of AnkH among many pathogenic obligate and facultative intracellular species of bacteria (12, 14) suggests its role in modulating an evolutionarily conserved eukaryotic process exploited by the various obligate and facultative intracellular pathogens that translocate the AnkH effector by the Dot/Icm T4SS. The results of a BLAST search (91) performed with the AnkH sequence show that, in addition to various *Legionella* species, homologous proteins are also found in *Gammaproteobacteria* species, *Coxiella* species, *Candidatus berkiella*, *Rickettsia* species, *Aquicella*, and Tatlockia micdadei. In most cases, the homologs contain all domains; in some cases, the C-terminal domain is partially or fully missing. All these homologs conserve the His-Asp-Cys catalytic triad residues, which are embedded in conserved patterns as follows: rG**H**a, **D/N**Rg and GN**C**SWANV (which are preserved down to the ∼30% levels of sequence identity with AnkH CPLD domain) (boldface highlighting represents the catalytic triad).

**FIG 7:**
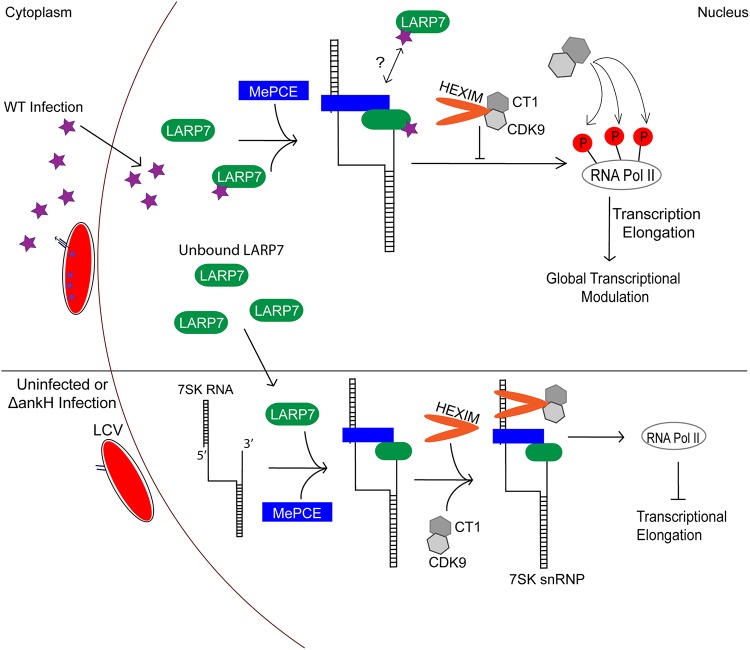
Working model of AnkH-LARP7 interaction. In uninfected cells or during Δ*ankH* mutant infection of HEK293T cells, formation of the 7SK snRNP begins when the 5′ methyl capping enzyme (MePCE) and LARP7 are recruited to the 7SK snRNA, forming the core of the 7SK snRNP. After core formation, the HEXIM1/2 dimers as well as the P-TEFb (Cdk9 and cyclin T1) kinase are recruited to complete the 7SK snRNP complex, which prevents transcription elongation by holding RNA polymerase (Pol) II in a paused state. During infection with WT L. pneumophila, AnkH is trafficked to the nucleus, where it interacts with a portion of available LARP7 in the cell. The interaction between AnkH and LARP7 results in a partial inhibition of the 7SK snRNP complex function, leading to enhanced transcriptional elongation by blocking the recruitment of HEXIM1/2 and P-TEFb. The remaining LARP7 present in the cell (the fraction that does not interact with AnkH) is available to interact with other components of the 7SK snRNP complex to pause transcription elongation by preventing P-TEFb from phosphorylating RNA polymerase 2, keeping the polymerase in a paused state. This balance between the pause and relief of the pause in transcriptional elongation results in transcriptional reprogramming within host cell that enhance permissiveness to L. pneumophila infection. There are likely other unidentified substrates of AnkH that could aid in this process or the could act independently of the interaction with LARP7. The effect on amoeba host transcription by AnkH may be different from that seen with human macrophages.

Since ARDs can potentially bind to multiple protein partners, it is likely that AnkH interacts with several other host proteins in addition to LARP7. Through the yeast two-hybrid screening, we identified six other host proteins with which AnkH might interact. Using co-IP, we confirmed that the host LARP7 component is an interaction partner for AnkH in HEK293T cells. Since residues within the β-hairpin loops of the ARDs are involved in binding to substrates, our data are consistent with these findings, as we have shown that substitution in β-hairpin loops of the ARD3 results in reduced binding of AnkH to LARP7, indicating that this loop is the more likely one to bind the LARP7 component of the 7SK snRNP complex, which controls pausing of Pol II at the initiation of transcriptional elongation (see model in Fig. 7) (63–65). Formation of the 7SK snRNP core complex (7SK, LARP7, and MePCE) enables recruitment of P-TEFb and HEXIM1/2 to the complex (64, 67, 70–73). Binding and sequestration of P-TEFb within the 7SK snRNP complex result in inhibition of its kinase activity and continuation of the pause in Pol II transcription elongation (66, 69, 74, 75). Various stimuli trigger the release of P-TEFb from the 7SK snRNP complex, leading to activation of its kinase activity and transition of Pol II into productive transcriptional elongation (76, 77). Our data indicate that LARP7 interacts with the β-hairpin loop of the third AR of AnkH, which impedes 7SKsnRNP complex formation, leading to productive transcriptional elongation by Pol II and host global transcriptional reprogramming. Our data show that there is an upregulation in pathways regulating transcription and immune responses in the presence of AnkH. In the absence of AnkH, however, there is an upregulation in pathways involved in vesicular trafficking, autophagy, and apoptosis. These observations support our findings with respect to the role of AnkH-LARP7 interaction in modulating the function of the 7SK snRNP complex in human macrophages, but the effect of AnkH on host global transcription is likely impacted by interaction of AnkH with other host targets. The range of pathogenic effectors that modulate host transcription machinery is limited, and the manipulation of the host 7SK snRNP complex via LARP7-AnkH interaction identifies a novel effector mechanism for host transcription control during infection. However, it is not known whether interaction of AnkH-LARP7 and potentially other host targets evolved during interaction of L. pneumophila with various protist hosts in the aquatic environment to modulate amoeba host-specific gene transcriptions that are highly conserved through evolution (12, 14, 32). It is highly possible that some of the transcriptional activity impacted by interaction of AnkH with LARP7 and other host targets in human macrophages may simply represent an evolutionary accident (12, 14, 32). Since knockdown of LARP7 resulted in a significant decrease in the intracellular replication of both the WT strain and the Δ*ankH* null mutant of L. pneumophila, it is likely that the AnkH-LARP7 interaction promotes transcription of genes involved in permissiveness to L. pneumophila in evolutionarily distant hosts. It was unexpected that LARP7 knockdown caused a significant decrease in intracellular replication of Δ*ankH* mutant. This could be explained by the hypothesis that AnkH does not interact with all LARP7 components available within a host cell, which could create a balance between the pausing of transcription elongation and relief of the pause in elongation that creates a favorable environment for L. pneumophila replication. When AnkH is deleted and LARP7 is knocked down, there is no longer a transcriptional balance, which results in the decrease in replication, likely as the result of alteration of many processes involved in permissiveness of the host cell to L. pneumophila.

The crystal structure revealed that AnkH contains four ARs and has two asparagine hydroxylation motifs located on the outer surface of the ARD in addition to the cysteine like protease pocket. Our data show that the predicted protease catalytic triad is essential for the function of AnkH, but we were not able to detect protease activity *in vitro* for AnkH purified from Escherichia coli. We speculate that the lack of detectable protease activity *in vitro* was likely due to the closed nature of the catalytic pocket of purified AnkH, suggesting a requirement of its binding to a substrate *in vivo* to open the pocket for catalysis. The ARDs are involved in protein-protein interactions by acting as a scaffold for protein binding. ARD-containing proteins can typically bind to one or more targets (38, 46). The ARDs containing multiple ankyrin repeats form crescent-like structures and contact their binding partners on the concave side that is formed from the inner short helices and the long β-hairpin/loop regions connecting the ankyrin repeats (92). Several residues on the putative target binding side of AnkH ARD that are located on the tips of the interrepeat loops are required for the function of AnkH in intracellular replication of L. pneumophila. These side chains are exposed to the solvent and aside from Tyr31 and Asn97 and are not in contact with the cysteine protease-like domain. Therefore, mutation of these residues likely disrupts the ability of AnkH to interact with LARP7 or other specific host targets. The mutation of Asn97 in particular led to a decrease in the ability of AnkH to bind LARP7, while mutations of other residues led to an enhanced binding, indicating that Asn97 is important for the AnkH-LARP7 interaction.

We have previously shown that AnkH is hydroxylated at N59 (55). We have also shown that the host FIH (factor inhibiting HIF [hypoxia-inducible factor]) asparagine hydroxylase localizes to the LCV and is involved in hydroxylating another L. pneumophila effector, AnkB (55). Asparagine hydroxylation of AnkB is also required for the function of the AnkB effector in intracellular replication of L. pneumophila (55). The asparagine hydroxylation motif is commonly found in ARDs and serves as a target sequence(s) for the FIH asparagine hydroxylase (93, 94), which is responsible for hydroxylating an asparagine residue within this motif (55). This hydroxylation can act as a molecular switch for protein-protein interactions by either inhibiting or strengthening the interaction (94–96). The N59 and N92 residues of ankH are located at the beginning of the loop connecting two neighboring ARDs. Our data show that the asparagine hydroxylation motifs are important for the function of AnkH in the intracellular replication. A possible explanation for the role of this modification is represented by the structure of the ankyrin domain of the mouse notch 1 with this modification (PDB ID: 2QC9) (97). The HIF-hydroxylated asparagine is located at a sharp bend of the backbone, and hydrogen bonds through the added hydroxyl with the aspartic acid side chain two residues back and located at the other corner of the bend. It has been suggested that this additional hydrogen bond might help to stabilize the loop in the ARD (97). Equivalent aspartic acids are found in AnkH at positions 57 and 90, two back from the asparagines. Therefore, a similar possibility of stabilization of the inter-ARD loops has to be considered for AnkH as a means to strengthen the interaction with its cellular target.

In summary, AnkH is targeted to the nucleus, where it interacts with LARP7 and likely other host targets, leading to reprograming of host transcription to promote intracellular bacterial growth. This is mediated, at least in part, by the effect of AnkH-LARP7 interaction and abolishment of interaction of LARP7 with critical subunits of the 7SK snRNP complex essential for its negative transcriptional elongation, leading to host global transcriptional reprogramming. The conservation of AnkH in intracellular pathogens harboring the Dot/Icm T4SS and its involvement in a conserved pathway support the idea of the AnkH-LARP7 interaction and its partial effect on reprogramming global host transcription, which is likely impacted by interaction of AnkH with other host targets. It is most likely the AnkH-dependent host transcriptional reprogramming the explains the unique consequences in various protist hosts compared to human macrophages. The crystal structure of AnkH shows that it contains an ARD with four ankyrin repeats containing two asparagine hydroxylation motifs, a cysteine protease-like domain, and a C-terminal domain of unknown function. Critical residues in the ARD and the cysteine protease-like domains identified from the structure are shown to be required for AnkH-LARP7 interaction and to function of AnkH in intracellular replication.

## MATERIALS AND METHODS

### Bacterial strains and cell culture.

L. pneumophila strain AA100/130b (BAA-74; American Type Culture Collection) and the isogeneic *dotA* and *ankH* mutants and complemented *ankH* mutants were grown on buffered charcoal yeast extract (BCYE) agar plates for 3 days at 37°C prior to use in infections, as described previously (38). E. coli strain DH5α was used for cloning purposes. Human monocyte-derived macrophages (hMDMs) were cultured using RPMI 1640 media (Gibco), as described previously (41). Maintenance of HEK293T cells was performed as previously described (41). All methods were carried out and approved in accordance to the University of Louisville Institutional Review Board guidelines, and blood donors gave informed consent as approved by the University of Louisville Institutional Review Board (IRB no. 04.0358).

### DNA manipulations.

DNA manipulations and restriction enzyme digestions were performed using standard procedures (41, 98). Restriction enzymes and T4 DNA ligase were purchased from NEB (Madison, WI). Plasmid preparations were performed with a PureLink HiPure Plasmid Maxiprep kit (Invitrogen). Purification of DNA fragments from agarose gels for subcloning was carried out with a QIAquick gel purification kit (Qiagen Inc., Valencia, CA). Generation of AnkH substitution mutants was achieved using primers listed in Table S1 and described previously (41, 54).

10.1128/mBio.01942-19.1 TABLE S1 Primers used in this study. Download Table S1, DOCX file, 0.02 MB. Copyright © 2019 Von Dwingelo et al. 2019 Von Dwingelo et al. This content is distributed under the terms of the Creative Commons Attribution 4.0 International license.

### Transfection of HEK293T cell.

HEK293T cells were grown to ∼70% confluence and plated onto poly-l-lysine-treated 24-well plates. Following 24 h of incubation, HEK293T cell monolayers were transfected with ∼2 μg of plasmid DNA/well by using polyethylenimine (Polysciences) and Opti-MEM (Gibco) for 24 h, as described previously (33, 54). The c-*myc*-LARP7 plasmid was a gift from B. Matija Peterlin, University of California, San Francisco.

### Confocal laser scanning microscopy.

Processing of transfected cells for confocal microscopy was performed as we described previously. Briefly, monolayers were permeabilized and fixed using 100% methanol held at –20°C for 5 min and were then blocked and labeled with mouse-anti-FLAG (Sigma) (1/200 dilution in 3% bovine serum albumin [BSA]–phosphate-buffered saline [PBS]) and rabbit-anti-Myc (Proteintech) (1/200 dilution in 3% BSA–PBS). Cells were counterlabeled with Alexa Fluor 488 anti-mouse antibody (Invitrogen) (1/4,000 dilution in 3% BSA–PBS), Alexa-Fluor 555 anti-rabbit antibody (Invitrogen) (1/4,000 dilution), and DAPI (4′,6-diamidino-2-phenylindole) to stain the nuclei. Monolayers were examined by confocal microscopy. A total of 100 cells for each replicate were counted to determine the presence or absence of localization.

### Intracellular replication.

The wild-type strain, the *dotA* and *ankH* isogenic mutants, and the complemented *ankH* mutants were grown on BCYE agar for 3 days at 37°C prior to infection and used to infect hMDMs. A total of 1 × 10^5^ host cells (hMDMs) per well were plated in 96-well plates and infected with L. pneumophila at a multiplicity of infection (MOI) of 10 for 1 h and then treated for 1 h with gentamicin to kill remaining extracellular bacteria. Over a 24-h time course, the host cells were lysed with sterile water and L. pneumophila CFU counts were determined by plating serial dilutions onto BCYE agar. Experiments were performed in triplicate.

### Yeast two-hybrid (Y2H) analysis.

A Matchmaker gold two-hybrid system (Clontech) was used to screen host proteins that interact with the AnkH protein per the instructions of the manufacturer. Full-length AnkH coding sequence was amplified, subjected to sequencing, cloned into the pGBKT7 bait vector (Clontech), and transformed into yeast strain AH109 (Clontech). A normalized universal human cDNA library in pGADT7 was purchased (Clontech) for use as potential prey targets. The library and bait containing AH109 were mated, and the resulting colonies were screened per the instructions of the manufacturer. Plasmids from positive clones were isolated using yeast lysis buffer and glass beads. Isolated prey plasmid and bait plasmid were used to cotransform the AH109 yeast strain. Transformants were selected by growing the yeast on SD medium lacking His, Leu, and Trp (SD-His/-Leu/-Trp) (Clontech). Colonies that tested positive were then transferred to SD-Ade/-His/-Leu/-Trp plates containing 5-bromo-4-chloro-3-indoxyl-α-d-galactopyranoside (X-α-Gal) (GoldBio). Blue colonies were selected for plasmid isolation. Isolated plasmids were then sequenced to identify the human genes.

### *In vivo* coimmunoprecipitation.

HEK293T cells were transfected with 3XFLAG-AnkH, BAP, and c-*myc*-LARP7 for 24 h and collected in lysis buffer, as described previously (79, 99). FLAG-tagged and myc-tagged proteins were immunoprecipitated by using anti-FLAG M2 magnetic beads (Sigma) or SureBeads protein G magnetic beads (Bio-Rad) cross-linked with anti-myc antibody (Proteintech).

### Antibodies and Western blot analysis.

Immunoprecipitated proteins were heated at 99°C for 5 min in sample buffer, separated by the use of 10.4% to 15% SDS-PAGE (Bio-Rad), and transferred onto a polyvinylidene difluoride (PVDF) (Bio-Rad) membrane, as described previously (99). Anti-Flag (Sigma) used at 1:1,000 dilution and anti-myc (60003-2-Ig) (Proteintech) used at 1:1,000 were incubated overnight in 8% milk at 4°C. Anti-LaminB (13435) (Cell Signaling) was used at a 1:1,000 dilution. Anti-HEXIM1 (15676-1-AP), anti-LARP7 (17067-1-AP), and anti-MePCE (14917-1-AP) were purchased from Proteintech and used at a 1:500 dilution. Anti-CDK9 (sc-13130) was purchased from Santa Cruz and used at a dilution of 1:200. Anti-cyclin T1 (sc-271348) was purchased from Santa Cruz and used at a dilution of 1:100. Goat anti-AnkH antiserum was produced at Cocalico Biologics and was used at a dilution of 1:100 (53, 54).

### RNA isolation, reverse transcription, and real-time PCR.

Total RNA was extracted using TRIzol reagent (Invitrogen). cDNA synthesis was performed with 1 μg of total RNA using an iScript cDNA synthesis kit (Bio-Rad) according to the manufacturer’s instructions. Endogenous mRNA levels were measured by real-time PCR analysis based on SYBR green detection (Fermentas) with a Bio-Rad MiniOpticon real-time PCR system.

### RNA-Seq.

Libraries were prepared using a TruSeq stranded mRNA LT sample prep kit (set A or set B) with poly(A) enrichment (Illumina). One microgram of sample (in a volume of 50 μl) were treated with RNA purification beads and denatured for 5 min at 65°C. The supernatant was then discarded, and the beads were washed with bead wash buffer. Captured polyadenylated RNA was eluted using elution buffer at 80°C for 2 min. mRNA was further purified in a second bead cleanup and was fragmented and primed during elution by adding 19.5 μg of Elute, Prime, Fragment, High mix to the beads and incubating the samples for 8 min at 94°C. After fragmentation, 17 μl of supernatant was removed from the beads and we proceeded immediately to synthesize first-strand cDNA.

Following the protocol, 8 μl of first-strand synthesis mix Act D and SuperScript II mix (Illumina) was added to each sample, and the mixture was heated on a thermocycler using preprogrammed thermal conditions. Once the reaction finished and its temperature reached 4°C, we immediately proceeded to second-strand cDNA synthesis.

Diluted end repair control and second-strand marking mix were added, and the reaction mixture was mixed well and incubated in a preheated thermocycler at 16°C for 1 h. The DNA was purified using Agencourt AMPure XP beads (Beckman). Finally, samples were eluted with resuspension buffer and 15 μl of elute was collected and stored at –20°C.

An A-Tailing control and an A-Tailing mix were added to the purified samples, and the samples were incubated on the preprogrammed thermal cycler. Once the incubation was done, we proceeded immediately to ligate adapters. Diluted ligation control, ligation mix, and barcodes were added, and the mixtures were incubated in a preheated thermocycler at 30°C for 10 min. Stop ligation buffer was immediately added to each sample, and the samples were mixed well. The ligated samples were then purified using Agencourt AMPure XP beads. We performed elutions with 50 μl of resuspension buffer, and the elute was purified for a second time using Agencourt AMPure XP beads. Afterward, the final elution, consisting of 20 μl of the elute, was collected and used for DNA enrichment. Samples were barcoded with Illumina TruSeq adapters as listed in Table S2. A complete list of the barcode sequences can be obtained from the Illumina support site (https://support.illumina.com/content/dam/illumina-support/documents/documentation/chemistry_documentation/experiment-design/illumina-adapter-sequences-1000000002694-11.pdf).

10.1128/mBio.01942-19.2 TABLE S2 Sample and barcode information. Download Table S2, DOCX file, 0.02 MB. Copyright © 2019 Von Dwingelo et al. 2019 Von Dwingelo et al. This content is distributed under the terms of the Creative Commons Attribution 4.0 International license.

PCR primer cocktail mix and PCR master mix were added to the samples, and the samples were incubated on a preprogrammed thermal cycler. The samples were then purified using Agencourt AMPure XP beads. Finally, 30 μl of eluted library was collected and stored at –20°C.

Libraries were validated by quality, where size, purity, and semiquantitation determinations were performed using an Agilent Bioanalyzer and an Agilent DNA 1000 kit. The final fragment size for all the samples was approximately 300 bp, which is the expected size according to the protocol. Libraries were also validated by quantity. Sequencing library quantitation was performed by qPCR using a KAPA library quantitation kit (KAPA Biosystems) for Illumina Platforms. The standard curve method was used for quantitation with 1 to 5 of the DNA standards that came with the kit.

Ten microliters of sample was transferred from the wells to a new Midi plate. We then normalized the concentrations of the libraries to 10 nM using Tris-HCl (10 mM, pH 8.5)–0.1% Tween 20. Five microliters of each sample was then transferred to be pooled into a new LowBind 1.5-ml microcentrifuge tube for a total library volume of 60 μl (pooled at 10 nM). Then, a 4 nM dilution was made from the 10 nM pooled library by dilution with Tris-HCl (10 mM, pH 8.5)–0.1% Tween 20.

A total volume of 1.3 ml of 1.8 pM denatured library is needed for sequencing using a v2 kit. A pooled 4 nM library was denatured by mixing with diluted NaOH and was incubated at room temperature for 5 min. Tris-HCl (200 mM, pH 7.0) was then added. The reaction mixture was diluted to 20 pM using a prechilled hybridization buffer. The 20 pM denatured library was further diluted to 1.8 pM using the same hybridization buffer. Before loading onto the reagent cartridge was performed, 1.3 μl of denatured 20 pM Phix control was added to the 1,299 μl of denatured 1.8 pM library to achieve a total volume of 1.3 ml for the sequencing run.

Sequencing was performed on an Illumina NextSeq 500 system at the University of Louisville Center for Genetics and Molecular Medicines (CGeMM) by the use of NextSeq 500/550 1 × 75-cycle high-output kit v2.

### RNAi knockdown.

Human LARP7 small interfering RNA (siRNA) lentivector against four LARP7 target sequences and scrambled siRNA GFP lentivector were used with pLEnti-P2A, pLenti-P2B, and Lentifectin to produce lentiviral particles per the protocol of the manufacturer (Applied Biological Materials, Inc.). Lentiviral particles were mixed with complete RPMI medium (Corning) containing 8 μg/ml Polybrene (Millipore). The mixture of virus and medium was added to wells at 50 μl mixture per 1 ml of cells and incubated for 24 h.

### *ankH* cloning and protein expression.

The *ankH* gene (Uniprot: Q5ZT65) from Legionella pneumophila strain Philadelphia 1 was cloned into vector pMCSG7, a derivative of vector pET-21a adapted for ligation-independent cloning (PMID: 18988021). This plasmid was then transformed into BL21(DE3) cells. The expressed protein contained a tobacco etch virus (TEV)-cleavable 6×His tag at the N terminus. For large-scale expression, a 15-ml overnight culture in LB was inoculated into 1 liter of Terrific broth medium (Bio Basic Inc., Markham, Ontario, Canada). The inoculated culture was grown at 37°C and was induced with 1 mM isopropyl β-d-1-thiogalactopyranoside when the optical density at 600 nm (OD_600_) reached 0.6, and the temperature was reduced to 18°C for overnight growth. The cells were harvested by centrifugation at 9,000 × *g* for 15 min.

For expression of the Se-methionine derivative, the cell pellet from 100 ml of overnight culture grown in LB media was inoculated into 1 liter of M9 minimal media. After shaking at 37°C was performed until the OD_600_ reached 0.6, a mixture of l-amino acids (100 mg of lysine, phenylalanine, and threonine; 50 mg of isoleucine, leucine, and valine) and 60 mg of Se-methionine was added to the culture. Protein expression was induced by adding 1 mM isopropyl β-d-1-thiogalactopyranoside after 15 min. The induced culture was grown overnight at 18°C, and the cells were harvested by centrifugation at 9,000 × *g* for 15 min.

### Protein purification.

The cell pellet was resuspended in lysis buffer (50 mM Tris-HCl buffer [pH 8.0], 10% glycerol, 0.1% Triton X). The cells were lysed in a cell disruptor (Constant Systems Ltd.). The cell debris was removed by centrifugation at 31,000 × *g* for 30 min. The resulting supernatant was applied to a 3 ml Talon cobalt metal-affinity column (Clontech). The column was washed with 5 column volumes of standard buffer (20 mM Tris [pH 8.0], 50 mM NaCl). A step gradient containing 100 mM and 200 mM imidazole in standard buffer was used to elute the His-tagged protein. Fractions containing AnkH were pooled and loaded on a Superdex 200 10/300 GL column (GE Healthcare) equilibrated with crystallization buffer (15 mM Tris-HCl [pH 8.0], 100 mM NaCl). AnkH-containing fractions were pooled and concentrated to 5 mg/ml in a Millipore centrifugal filter with a molecular weight cutoff of 10,000 Da for crystallization trials. The concentration was measured using a NanoDrop UV spectrophotometer (Thermo Scientific) and an extinction coefficient of 70,250 for AnkH, calculated by the use of the ProtParam tool (100).

### Protein crystallization and data collection.

Initial crystals were obtained by screening and were optimized using the 24-well plate format. The best crystals were obtained by the hanging-drop method by mixing 1 μl of protein solution and 1 μl of reservoir solution containing 1.0 M ammonium tartrate dibasic (pH 7.0). The drop was incubated over a 0.5-ml reservoir solution. The crystals were cryoprotected in solution containing 70% of reservoir solution and 30% glycerol. Crystals were flash cooled in liquid nitrogen, and diffraction data were collected at the 08ID and 08BM beamlines at the Canadian Light Source. Data were processed and scaled with XDS. The same procedure was followed for the Se-methionine-labeled derivative.

### Structure determination.

The native and SeMet data set were indexed, integrated, and scaled using Program HKL3000 (101). Experimental phases were obtained by the single-wavelength anomalous dispersion (SAD) method, and the structure was solved using Program HKL3000. The autobuilt model from HKL3000 was ∼90% complete, and the remaining 10% of the molecule was built manually using Coot software (PMID: 20383002). The refinement was done using the Phenix program suite (102). The model contained residues 1 to 461 and was refined to *R*_work_ = 0.172 and *R*_free_ = 0.210. The geometry was validated with the program MolProbity (103). The pertinent details of data collection and refinement are listed in Table S5. The coordinates and structure factors were deposited with the Protein Data Bank with the code 6MCA. The crystal structure was modeled using Chimera software (University of California, Sam Francisco [UCSF]), and structure similarity to other peptidases was determined using the MEROPS peptidase database (89).

10.1128/mBio.01942-19.5 TABLE S5 Data collection and refinement. Download Table S5, DOCX file, 0.02 MB. Copyright © 2019 Von Dwingelo et al. 2019 Von Dwingelo et al. This content is distributed under the terms of the Creative Commons Attribution 4.0 International license.

### Statistical analysis.

All experiments were performed using at least three independent biological repeats, and the data shown are representative of results from one experiment. To analyze for statistically significant differences among three sets of data, the two-tailed Student's *t* test was used, and the *P* value was obtained.

### Data availability.

Atomic coordinates and diffraction data for the structure have been deposited with PDB (https://www.wwpdb.org/) under PDB ID 6MCA. The RNA-Seq data have been submitted to the GEO database (https://www.ncbi.nlm.nih.gov/geo/), accession no. GSE135803.

## References

[1] Abu Khweek A, Amer AO 2018 Factors mediating environmental biofilm formation by Legionella pneumophila. Front Cell Infect Microbiol 8:38. doi:10.3389/fcimb.2018.00038. 29535972 PMC5835138

[2] Fields BS 1996 The molecular ecology of legionellae. Trends Microbiol 4:286–290. doi:10.1016/0966-842X(96)10041-X. 8829338

[3] Horwitz MA, Silverstein SC 1980 Legionnaires’ disease bacterium (*Legionella pneumophila*) multiplies intracellularly in human monocytes. J Clin Invest 66:441–450. doi:10.1172/JCI109874. 7190579 PMC371671

[4] Rowbotham TJ 1980 Preliminary report on the pathogenicity of *Legionella pneumophila* for freshwater and soil amoebae. J Clin Pathol 33:1179–1183. doi:10.1136/jcp.33.12.1179. 7451664 PMC1146371

[5] Swart AL, Harrison CF, Eichinger L, Steinert M, Hilbi H 2018 Acanthamoeba and dictyostelium as cellular models for Legionella infection. Front Cell Infect Microbiol 8:61. doi:10.3389/fcimb.2018.00061. 29552544 PMC5840211

[6] Abu Kwaik Y, Gao L-Y, Stone BJ, Venkataraman C, Harb OS 1998 Invasion of protozoa by *Legionella pneumophila* and its role in bacterial ecology and pathogenesis. Appl Environ Microbiol 64:3127–3133. 9726849 10.1128/aem.64.9.3127-3133.1998 PMC106699

[7] Fields BS, Benson RF, Besser RE 2002 Legionella and Legionnaires’ disease: 25 years of investigation. Clin Microbiol Rev 15:506–526. doi:10.1128/cmr.15.3.506-526.2002. 12097254 PMC118082

[8] Garcia MT, Jones S, Pelaz C, Millar RD, Abu Kwaik Y 2007 Acanthamoeba polyphaga resuscitates viable non-culturable Legionella pneumophila after disinfection. Environ Microbiol 9:1267–1277. doi:10.1111/j.1462-2920.2007.01245.x. 17472639

[9] Best AM, Abu Kwaik Y 2019 Evasion of phagotrophic predation by protist hosts and innate immunity of metazoan hosts by Legionella pneumophila. Cell Microbiol 21:e12971. doi:10.1111/cmi.12971. 30370624 PMC6296878

[10] Boamah DK, Zhou G, Ensminger AW, O'Connor TJ 2017 From many hosts, one accidental pathogen: the diverse protozoan hosts of Legionella. Front Cell Infect Microbiol 7:477. doi:10.3389/fcimb.2017.00477. 29250488 PMC5714891

[11] Wu H-Y, Yan H, Zheng M-L, Sun M-M, Wang Q, Hu C-M, Zhan X-Y, Yuan M-G, Qu P-H, Hu C-H 2019 Legionella qingyii sp. nov., isolated from water samples in China. Int J Syst Evol Microbiol 69:2017–2022. doi:10.1099/ijsem.0.003421. 31063123

[12] Burstein D, Amaro F, Zusman T, Lifshitz Z, Cohen O, Gilbert JA, Pupko T, Shuman HA, Segal G 2016 Genomic analysis of 38 Legionella species identifies large and diverse effector repertoires. Nat Genet 48:167–175. doi:10.1038/ng.3481. 26752266 PMC5050043

[13] Gomez-Valero L, Rusniok C, Rolando M, Neou M, Dervins-Ravault D, Demirtas J, Rouy Z, Moore RJ, Chen H, Petty NK, Jarraud S, Etienne J, Steinert M, Heuner K, Gribaldo S, Médigue C, Glöckner G, Hartland EL, Buchrieser C 2014 Comparative analyses of Legionella species identifies genetic features of strains causing Legionnaires’ disease. Genome Biol 15:505. doi:10.1186/PREACCEPT-1086350395137407. 25370836 PMC4256840

[14] Gomez-Valero L, Rusniok C, Carson D, Mondino S, Pérez-Cobas AE, Rolando M, Pasricha S, Reuter S, Demirtas J, Crumbach J, Descorps-Declere S, Hartland EL, Jarraud S, Dougan G, Schroeder GN, Frankel G, Buchrieser C 2019 More than 18,000 effectors in the *Legionella* genus genome provide multiple, independent combinations for replication in human cells. Proc Natl Acad Sci U S A 116:2265–2273. doi:10.1073/pnas.1808016116. 30659146 PMC6369783

[15] Yu VL, Plouffe JF, Pastoris MC, Stout JE, Schousboe M, Widmer A, Summersgill J, File T, Heath CM, Paterson DL, Chereshsky A 2002 Distribution of Legionella species and serogroups isolated by culture in patients with sporadic community-acquired legionellosis: an international collaborative survey. J Infect Dis 186:127–128. doi:10.1086/341087. 12089674

[16] Isberg RR, O'Connor TJ, Heidtman M 2009 The Legionella pneumophila replication vacuole: making a cozy niche inside host cells. Nat Rev Microbiol 7:13–24. doi:10.1038/nrmicro1967. 19011659 PMC2631402

[17] Luo ZQ 2011 Legionella secreted effectors and innate immune responses. Cell Microbiol doi:10.1111/j.1462-5822.2011.01713.x. PMC3538141 21985602

[18] Molmeret M, Horn M, Wagner M, Santic M, Abu Kwaik Y 2005 Amoebae as training grounds for intracellular bacterial pathogens. Appl Environ Microbiol 71:20–28. doi:10.1128/AEM.71.1.20-28.2005. 15640165 PMC544274

[19] Oliva G, Sahr T, Buchrieser C 2018 The life cycle of L. pneumophila: cellular differentiation is linked to virulence and metabolism. Front Cell Infect Microbiol 8:3. doi:10.3389/fcimb.2018.00003. 29404281 PMC5780407

[20] Shin S, Roy CR 2008 Host cell processes that influence the intracellular survival of Legionella pneumophila. Cell Microbiol 10:1209–1220. doi:10.1111/j.1462-5822.2008.01145.x. 18363881

[21] Coers J, Monahan C, Roy CR 1999 Modulation of phagosome biogenesis by *Legionella pneumophila* creates an organelle permissive for intracellular growth. Nat Cell Biol 1:451–453. doi:10.1038/15687. 10559990

[22] Kagan JC, Roy CR 2002 Legionella phagosomes intercept vesicular traffic from endoplasmic reticulum exit sites. Nat Cell Biol 4:945–954. doi:10.1038/ncb883. 12447391

[23] Barlocher K, Welin A, Hilbi H 2017 Formation of the Legionella replicative compartment at the crossroads of retrograde trafficking. Front Cell Infect Microbiol 7:482. doi:10.3389/fcimb.2017.00482. 29226112 PMC5706426

[24] Ghosh S, O'Connor TJ 2017 Beyond paralogs: the multiple layers of redundancy in bacterial pathogenesis. Front Cell Infect Microbiol 7:467. doi:10.3389/fcimb.2017.00467. 29188194 PMC5694747

[25] Steiner B, Swart AL, Welin A, Weber S, Personnic N, Kaech A, Freyre C, Ziegler U, Klemm RW, Hilbi H 2017 ER remodeling by the large GTPase atlastin promotes vacuolar growth of Legionella pneumophila. EMBO Rep 18:1817. doi:10.15252/embr.201743903. 28835546 PMC5623866

[26] Ensminger AW 2016 Legionella pneumophila, armed to the hilt: justifying the largest arsenal of effectors in the bacterial world. Curr Opin Microbiol 29:74–80. doi:10.1016/j.mib.2015.11.002. 26709975

[27] Schroeder GN 2018 The toolbox for uncovering the functions of Legionella Dot/Icm type IVb secretion system effectors: current state and future directions. Front Cell Infect Microbiol 7:528. doi:10.3389/fcimb.2017.00528. 29354599 PMC5760550

[28] Manske C, Hilbi H 2014 Metabolism of the vacuolar pathogen Legionella and implications for virulence. Front Cell Infect Microbiol 4:125. doi:10.3389/fcimb.2014.00125. 25250244 PMC4158876

[29] Qiu J, Luo Z-Q 2017 Legionella and Coxiella effectors: strength in diversity and activity. Nat Rev Microbiol 15:591–605. doi:10.1038/nrmicro.2017.67. 28713154

[30] Qiu J, Luo Z-Q 2017 Hijacking of the host ubiquitin network by Legionella pneumophila. Front Cell Infect Microbiol 7:487. doi:10.3389/fcimb.2017.00487. 29376029 PMC5770618

[31] O’Connor TJ, Adepoju Y, Boyd D, Isberg RR 2011 Minimization of the Legionella pneumophila genome reveals chromosomal regions involved in host range expansion. Proc Natl Acad Sci U S A 108:14733–14740. doi:10.1073/pnas.1111678108. 21873199 PMC3169125

[32] Best A, Abu Kwaik Y 2018 Evolution of the arsenal of Legionella pneumophila effectors to modulate protist hosts. mBio 9:e01313-18. doi:10.1128/mBio.01313-18. 30301851 PMC6178616

[33] Al-Quadan T, Price C, Abu Kwaik Y 2012 Exploitation of evolutionarily conserved amoeba and mammalian processes by *Legionella*. Trends Microbiol 20:299–306. doi:10.1016/j.tim.2012.03.005. 22494803 PMC3603140

[34] Cazalet C, Rusniok C, Bruggemann H, Zidane N, Magnier A, Ma L, Tichit M, Jarraud S, Bouchier C, Vandenesch F, Kunst F, Etienne J, Glaser P, Buchrieser C 2004 Evidence in the Legionella pneumophila genome for exploitation of host cell functions and high genome plasticity. Nat Genet 36:1165–1173. doi:10.1038/ng1447. 15467720

[35] de Felipe KS, Pampou S, Jovanovic OS, Pericone CD, Ye SF, Kalachikov S, Shuman HA 2005 Evidence for acquisition of Legionella type IV secretion substrates via interdomain horizontal gene transfer. J Bacteriol 187:7716–7726. doi:10.1128/JB.187.22.7716-7726.2005. 16267296 PMC1280299

[36] Franco IS, Shuman HA, Charpentier X 2009 The perplexing functions and surprising origins of Legionella pneumophila type IV secretion effectors. Cell Microbiol 11:1435–1443. doi:10.1111/j.1462-5822.2009.01351.x. 19563462

[37] Gomez-Valero L, Rusniok C, Cazalet C, Buchrieser C 2011 Comparative and functional genomics of legionella identified eukaryotic like proteins as key players in host-pathogen interactions. Front Microbiol 2:208. doi:10.3389/fmicb.2011.00208. 22059087 PMC3203374

[38] Al-Khodor S, Price CT, Habyarimana F, Kalia A, Abu Kwaik Y 2008 A Dot/Icm-translocated ankyrin protein of Legionella pneumophila is required for intracellular proliferation within human macrophages and protozoa. Mol Microbiol 70:908–923. doi:10.1111/j.1365-2958.2008.06453.x. 18811729 PMC3064707

[39] Ivanov SS, Charron G, Hang HC, Roy CR 2010 Lipidation by the host prenyltransferase machinery facilitates membrane localization of Legionella pneumophila effector proteins. J Biol Chem 285:34686–34698. doi:10.1074/jbc.M110.170746. 20813839 PMC2966084

[40] Kubori T, Hyakutake A, Nagai H 2008 Legionella translocates an E3 ubiquitin ligase that has multiple U-boxes with distinct functions. Mol Microbiol 67:1307–1319. doi:10.1111/j.1365-2958.2008.06124.x. 18284575

[41] Price CT, Al-Khodor S, Al-Quadan T, Santic M, Habyarimana F, Kalia A, Kwaik YA 2009 Molecular mimicry by an F-box effector of Legionella pneumophila hijacks a conserved polyubiquitination machinery within macrophages and protozoa. PLoS Pathog 5:e1000704. doi:10.1371/journal.ppat.1000704. 20041211 PMC2790608

[42] Zamboni DS, Kobayashi KS, Kohlsdorf T, Ogura Y, Long EM, Vance RE, Kuida K, Mariathasan S, Dixit VM, Flavell RA, Dietrich WF, Roy CR 2006 The Birc1e cytosolic pattern-recognition receptor contributes to the detection and control of Legionella pneumophila infection. Nat Immunol 7:318–325. doi:10.1038/ni1305. 16444259

[43] Binz HK, Kohl A, Pluckthun A, Grutter MG 2006 Crystal structure of a consensus-designed ankyrin repeat protein: implications for stability. Proteins 65:280–284. doi:10.1002/prot.20930. 16493627

[44] Bork P 1993 Hundreds of ankyrin-like repeats in functionally diverse proteins: mobile modules that cross phyla horizontally? Proteins 17:363–374. doi:10.1002/prot.340170405. 8108379

[45] Kohl A, Binz HK, Forrer P, Stumpp MT, Pluckthun A, Grutter MG 2003 Designed to be stable: crystal structure of a consensus ankyrin repeat protein. Proc Natl Acad Sci U S A 100:1700–1705. doi:10.1073/pnas.0337680100. 12566564 PMC149896

[46] Li J, Mahajan A, Tsai MD 2006 Ankyrin repeat: a unique motif mediating protein-protein interactions. Biochemistry 45:15168–15178. doi:10.1021/bi062188q. 17176038

[47] Mosavi LK, Minor DL Jr, Peng ZY 2002 Consensus-derived structural determinants of the ankyrin repeat motif. Proc Natl Acad Sci U S A 99:16029–16034. doi:10.1073/pnas.252537899. 12461176 PMC138559

[48] Yu H, Kohl A, Binz HK, Pluckthun A, Grutter MG, van Gunsteren WF 2006 Molecular dynamics study of the stabilities of consensus designed ankyrin repeat proteins. Proteins 65:285–295. doi:10.1002/prot.20991. 16948156

[49] Sedgwick SG, Smerdon SJ 1999 The ankyrin repeat: a diversity of interactions on a common structural framework. Trends Biochem Sci 24:311–316. doi:10.1016/S0968-0004(99)01426-7. 10431175

[50] Islam Z, Nagampalli RSK, Fatima MT, Ashraf GM 2018 New paradigm in ankyrin repeats: beyond protein-protein interaction module. Int J Biol Macromol 109:1164–1173. doi:10.1016/j.ijbiomac.2017.11.101. 29157912

[51] Al-Khodor S, Price CT, Kalia A, Abu Kwaik Y 2010 Functional diversity of ankyrin repeats in microbial proteins. Trends Microbiol 18:132–139. doi:10.1016/j.tim.2009.11.004. 19962898 PMC2834824

[52] Pan X, Luhrmann A, Satoh A, Laskowski-Arce MA, Roy CR 2008 Ankyrin repeat proteins comprise a diverse family of bacterial type IV effectors. Science 320:1651–1654. doi:10.1126/science.1158160. 18566289 PMC2514061

[53] Habyarimana F, Al-Khodor S, Kalia A, Graham JE, Price CT, Garcia MT, Kwaik YA 2008 Role for the ankyrin eukaryotic-like genes of Legionella pneumophila in parasitism of protozoan hosts and human macrophages. Environ Microbiol 10:1460–1474. doi:10.1111/j.1462-2920.2007.01560.x. 18279343

[54] Habyarimana F, Price CT, Santic M, Al-Khodor S, Kwaik YA 2010 Molecular characterization of the Dot/Icm-translocated AnkH and AnkJ eukaryotic-like effectors of Legionella pneumophila. Infect Immun 78:1123–1134. doi:10.1128/IAI.00913-09. 20028808 PMC2825944

[55] Price C, Merchant M, Jones S, Best A, Von Dwingelo J, Lawrenz MB, Alam N, Schueler-Furman O, Kwaik YA 2017 Host FIH-mediated asparaginyl hydroxylation of translocated Legionella pneumophila effectors. Front Cell Infect Microbiol 7:54. doi:10.3389/fcimb.2017.00054. 28321389 PMC5337513

[56] Belyi Y, Niggeweg R, Opitz B, Vogelsgesang M, Hippenstiel S, Wilm M, Aktories K 2006 Legionella pneumophila glucosyltransferase inhibits host elongation factor 1A. Proc Natl Acad Sci U S A 103:16953–16958. doi:10.1073/pnas.0601562103. 17068130 PMC1636560

[57] Belyi Y, Tabakova I, Stahl M, Aktories K 2008 Lgt: a family of cytotoxic glucosyltransferases produced by Legionella pneumophila. J Bacteriol 190:3026–3035. doi:10.1128/JB.01798-07. 18281405 PMC2293231

[58] Fontana MF, Banga S, Barry KC, Shen X, Tan Y, Luo ZQ, Vance RE 2011 Secreted bacterial effectors that inhibit host protein synthesis are critical for induction of the innate immune response to virulent Legionella pneumophila. PLoS Pathog 7:e1001289. doi:10.1371/journal.ppat.1001289. 21390206 PMC3040669

[59] Shen X, Banga S, Liu Y, Xu L, Gao P, Shamovsky I, Nudler E, Luo ZQ 2009 Targeting eEF1A by a Legionella pneumophila effector leads to inhibition of protein synthesis and induction of host stress response. Cell Microbiol 11:911–926. doi:10.1111/j.1462-5822.2009.01301.x. 19386084 PMC2967282

[60] Li T, Lu Q, Wang G, Xu H, Huang H, Cai T, Kan B, Ge J, Shao F 2013 SET-domain bacterial effectors target heterochromatin protein 1 to activate host rDNA transcription. EMBO Rep 14:733–740. doi:10.1038/embor.2013.86. 23797873 PMC3736128

[61] Rolando M, Sanulli S, Rusniok C, Gomez-Valero L, Bertholet C, Sahr T, Margueron R, Buchrieser C 2013 Legionella pneumophila effector RomA uniquely modifies host chromatin to repress gene expression and promote intracellular bacterial replication. Cell Host Microbe 13:395–405. doi:10.1016/j.chom.2013.03.004. 23601102

[62] Lee PC, Machner MP 2018 The Legionella effector kinase LegK7 hijacks the host Hippo pathway to promote infection. Cell Host Microbe 24:429–438.e6. doi:10.1016/j.chom.2018.08.004. 30212651 PMC7343393

[63] Price DH 2000 P-TEFb, a cyclin-dependent kinase controlling elongation by RNA polymerase II. Mol Cell Biol 20:2629–2634. doi:10.1128/mcb.20.8.2629-2634.2000. 10733565 PMC85478

[64] Zhou Q, Li T, Price DH 2012 RNA polymerase II elongation control. Annu Rev Biochem 81:119–143. doi:10.1146/annurev-biochem-052610-095910. 22404626 PMC4273853

[65] Uchikawa E, Natchiar KS, Han X, Proux F, Roblin P, Zhang E, Durand A, Klaholz BP, Dock-Bregeon AC 2015 Structural insight into the mechanism of stabilization of the 7SK small nuclear RNA by LARP7. Nucleic Acids Res 43:3373–3388. doi:10.1093/nar/gkv173. 25753663 PMC4381077

[66] Barboric M, Lenasi T, Chen H, Johansen EB, Guo S, Peterlin BM 2009 7SK snRNP/P-TEFb couples transcription elongation with alternative splicing and is essential for vertebrate development. Proc Natl Acad Sci U S A 106:7798–7803. doi:10.1073/pnas.0903188106. 19416841 PMC2683122

[67] He N, Jahchan NS, Hong E, Li Q, Bayfield MA, Maraia RJ, Luo K, Zhou Q 2008 A La-related protein modulates 7SK snRNP integrity to suppress P-TEFb-dependent transcriptional elongation and tumorigenesis. Mol Cell 29:588–599. doi:10.1016/j.molcel.2008.01.003. 18249148 PMC6239424

[68] Muniz L, Egloff S, Kiss T 2013 RNA elements directing in vivo assembly of the 7SK/MePCE/Larp7 transcriptional regulatory snRNP. Nucleic Acids Res 41:4686–4698. doi:10.1093/nar/gkt159. 23471002 PMC3632141

[69] Xue Y, Yang Z, Chen R, Zhou Q 2010 A capping-independent function of MePCE in stabilizing 7SK snRNA and facilitating the assembly of 7SK snRNP. Nucleic Acids Res 38:360–369. doi:10.1093/nar/gkp977. 19906723 PMC2811026

[70] Guo J, Price DH 2013 RNA polymerase II transcription elongation control. Chem Rev 113:8583–8603. doi:10.1021/cr400105n. 23919563 PMC4294624

[71] Jeronimo C, Forget D, Bouchard A, Li Q, Chua G, Poitras C, Therien C, Bergeron D, Bourassa S, Greenblatt J, Chabot B, Poirier GG, Hughes TR, Blanchette M, Price DH, Coulombe B 2007 Systematic analysis of the protein interaction network for the human transcription machinery reveals the identity of the 7SK capping enzyme. Mol Cell 27:262–274. doi:10.1016/j.molcel.2007.06.027. 17643375 PMC4498903

[72] Michels AA, Fraldi A, Li Q, Adamson TE, Bonnet F, Nguyen VT, Sedore SC, Price JP, Price DH, Lania L, Bensaude O 2004 Binding of the 7SK snRNA turns the HEXIM1 protein into a P-TEFb (CDK9/cyclin T) inhibitor. EMBO J 23:2608–2619. doi:10.1038/sj.emboj.7600275. 15201869 PMC449783

[73] Yik JH, Chen R, Nishimura R, Jennings JL, Link AJ, Zhou Q 2003 Inhibition of P-TEFb (CDK9/cyclin T) kinase and RNA polymerase II transcription by the coordinated actions of HEXIM1 and 7SK snRNA. Mol Cell 12:971–982. doi:10.1016/S1097-2765(03)00388-5. 14580347

[74] Nguyen VT, Kiss T, Michels AA, Bensaude O 2001 7SK small nuclear RNA binds to and inhibits the activity of CDK9/cyclin T complexes. Nature 414:322–325. doi:10.1038/35104581. 11713533

[75] Yang Z, Zhu Q, Luo K, Zhou Q 2001 The 7SK small nuclear RNA inhibits the CDK9/cyclin T1 kinase to control transcription. Nature 414:317–322. doi:10.1038/35104575. 11713532

[76] Perumal K, Sinha K, Henning D, Reddy R 2001 Purification, characterization, and cloning of the cDNA of human signal recognition particle RNA 3’-adenylating enzyme. J Biol Chem 276:21791–21796. doi:10.1074/jbc.M101905200. 11287430

[77] Sinha KM, Gu J, Chen Y, Reddy R 1998 Adenylation of small RNAs in human cells. Development of a cell-free system for accurate adenylation on the 3’-end of human signal recognition particle RNA. J Biol Chem 273:6853–6859. doi:10.1074/jbc.273.12.6853. 9506988

[78] Peterlin BM, Price DH 2006 Controlling the elongation phase of transcription with P-TEFb. Mol Cell 23:297–305. doi:10.1016/j.molcel.2006.06.014. 16885020

[79] Price CT, Al-Quadan T, Santic M, Jones SC, Abu Kwaik Y 2010 Exploitation of conserved eukaryotic host cell farnesylation machinery by an F-box effector of Legionella pneumophila. J Exp Med 207:1713–1726. doi:10.1084/jem.20100771. 20660614 PMC2916131

[80] Krueger BJ, Jeronimo C, Roy BB, Bouchard A, Barrandon C, Byers SA, Searcey CE, Cooper JJ, Bensaude O, Cohen EA, Coulombe B, Price DH 2008 LARP7 is a stable component of the 7SK snRNP while P-TEFb, HEXIM1 and hnRNP A1 are reversibly associated. Nucleic Acids Res 36:2219–2229. doi:10.1093/nar/gkn061. 18281698 PMC2367717

[81] Holm L, Rosenstrom P 2010 Dali server: conservation mapping in 3D. Nucleic Acids Res 38:W545–W549. doi:10.1093/nar/gkq366. 20457744 PMC2896194

[82] Abendroth U, Adlung N, Otto A, Gruneisen B, Becher D, Bonas U 2017 Identification of new protein-coding genes with a potential role in the virulence of the plant pathogen Xanthomonas euvesicatoria. BMC Genomics 18:625. doi:10.1186/s12864-017-4041-7. 28814272 PMC5559785

[83] Chosed R, Tomchick DR, Brautigam CA, Mukherjee S, Negi VS, Machius M, Orth K 2007 Structural analysis of Xanthomonas XopD provides insights into substrate specificity of ubiquitin-like protein proteases. J Biol Chem 282:6773–6782. doi:10.1074/jbc.M608730200. 17204475

[84] Pruneda JN, Durkin CH, Geurink PP, Ovaa H, Santhanam B, Holden DW, Komander D 2016 The molecular basis for ubiquitin and ubiquitin-like specificities in bacterial effector proteases. Mol Cell 63:261–276. doi:10.1016/j.molcel.2016.06.015. 27425412 PMC4961225

[85] Yang A, Pantoom S, Wu YW 2017 Elucidation of the anti-autophagy mechanism of the Legionella effector RavZ using semisynthetic LC3 proteins. Elife 6:e23905. doi:10.7554/eLife.23905. 28395732 PMC5388539

[86] Horenkamp FA, Kauffman KJ, Kohler LJ, Sherwood RK, Krueger KP, Shteyn V, Roy CR, Melia TJ, Reinisch KM 2015 The Legionella anti-autophagy effector RavZ targets the autophagosome via PI3P- and curvature-sensing motifs. Dev Cell 34:569–576. doi:10.1016/j.devcel.2015.08.010. 26343456 PMC4594837

[87] Finn RD, Attwood TK, Babbitt PC, Bateman A, Bork P, Bridge AJ, Chang HY, Dosztanyi Z, El-Gebali S, Fraser M, Gough J, Haft D, Holliday GL, Huang H, Huang X, Letunic I, Lopez R, Lu S, Marchler-Bauer A, Mi H, Mistry J, Natale DA, Necci M, Nuka G, Orengo CA, Park Y, Pesseat S, Piovesan D, Potter SC, Rawlings ND, Redaschi N, Richardson L, Rivoire C, Sangrador-Vegas A, Sigrist C, Sillitoe I, Smithers B, Squizzato S, Sutton G, Thanki N, Thomas PD, Tosatto SC, Wu CH, Xenarios I, Yeh LS, Young SY, Mitchell AL 2017 InterPro in 2017-beyond protein family and domain annotations. Nucleic Acids Res 45:D190–D199. doi:10.1093/nar/gkw1107. 27899635 PMC5210578

[88] Rawlings ND, Barrett AJ, Finn R 2016 Twenty years of the MEROPS database of proteolytic enzymes, their substrates and inhibitors. Nucleic Acids Res 44:D343–D350. doi:10.1093/nar/gkv1118. 26527717 PMC4702814

[89] Rawlings ND, Barrett AJ, Thomas PD, Huang X, Bateman A, Finn RD 2018 The MEROPS database of proteolytic enzymes, their substrates and inhibitors in 2017 and a comparison with peptidases in the PANTHER database. Nucleic Acids Res 46:D624–D632. doi:10.1093/nar/gkx1134. 29145643 PMC5753285

[90] Du Toit A 2019 The effector repertoire of Legionella. Nat Rev Microbiol 17:126. doi:10.1038/s41579-019-0155-z. 30710106

[91] Altschul SF, Madden TL, Schaffer AA, Zhang J, Zhang Z, Miller W, Lipman DJ 1997 Gapped BLAST and PSI-BLAST: a new generation of protein database search programs. Nucleic Acids Res 25:3389–3402. doi:10.1093/nar/25.17.3389. 9254694 PMC146917

[92] Mosavi LK, Cammett TJ, Desrosiers DC, Peng ZY 2004 The ankyrin repeat as molecular architecture for protein recognition. Protein Sci 13:1435–1448. doi:10.1110/ps.03554604. 15152081 PMC2279977

[93] Lando D, Peet DJ, Whelan DA, Gorman JJ, Whitelaw ML 2002 Asparagine hydroxylation of the HIF transactivation domain a hypoxic switch. Science 295:858–861. doi:10.1126/science.1068592. 11823643

[94] Mahon PC, Hirota K, Semenza GL 2001 FIH-1: a novel protein that interacts with HIF-1α and VHL to mediate repression of HIF-1 transcriptional activity. Genes Dev 15:2675–2686. doi:10.1101/gad.924501. 11641274 PMC312814

[95] Hewitson KS, McNeill LA, Riordan MV, Tian YM, Bullock AN, Welford RW, Elkins JM, Oldham NJ, Bhattacharya S, Gleadle JM, Ratcliffe PJ, Pugh CW, Schofield CJ 2002 Hypoxia-inducible factor (HIF) asparagine hydroxylase is identical to factor inhibiting HIF (FIH) and is related to the cupin structural family. J Biol Chem 277:26351–26355. doi:10.1074/jbc.C200273200. 12042299

[96] Yang M, Ge W, Chowdhury R, Claridge TD, Kramer HB, Schmierer B, McDonough MA, Gong L, Kessler BM, Ratcliffe PJ, Coleman ML, Schofield CJ 2011 Asparagine and aspartate hydroxylation of the cytoskeletal ankyrin family is catalyzed by factor-inhibiting hypoxia-inducible factor. J Biol Chem 286:7648–7660. doi:10.1074/jbc.M110.193540. 21177872 PMC3045019

[97] Coleman ML, McDonough MA, Hewitson KS, Coles C, Mecinovic J, Edelmann M, Cook KM, Cockman ME, Lancaster DE, Kessler BM, Oldham NJ, Ratcliffe PJ, Schofield CJ 2007 Asparaginyl hydroxylation of the Notch ankyrin repeat domain by factor inhibiting hypoxia-inducible factor. J Biol Chem 282:24027–24038. doi:10.1074/jbc.M704102200. 17573339

[98] Stone BJ, Abu Kwaik Y 1998 Expression of multiple pili by *Legionella pneumophila*: identification and characterization of a type IV pilin gene and its role in adherence to mammalian and protozoan cells. Infect Immun 66:1768–1775. 9529112 10.1128/iai.66.4.1768-1775.1998 PMC108119

[99] Bruckert WM, Abu Kwaik Y 2016 Lysine11-linked polyubiquitination of the AnkB F-box effector of Legionella pneumophila. Infect Immun 84:99–107. doi:10.1128/IAI.01165-15. 26483404 PMC4694019

[100] Wilkins MR, Gasteiger E, Bairoch A, Sanchez JC, Williams KL, Appel RD, Hochstrasser DF 1999 Protein identification and analysis tools in the ExPASy server. Methods Mol Biol 112:531–552. 10027275 10.1385/1-59259-584-7:531

[101] Minor W, Cymborowski M, Otwinowski Z, Chruszcz M 2006 HKL-3000: the integration of data reduction and structure solution–from diffraction images to an initial model in minutes. Acta Crystallogr D Biol Crystallogr 62:859–866. doi:10.1107/S0907444906019949. 16855301

[102] Adams PD, Afonine PV, Bunkoczi G, Chen VB, Davis IW, Echols N, Headd JJ, Hung LW, Kapral GJ, Grosse-Kunstleve RW, McCoy AJ, Moriarty NW, Oeffner R, Read RJ, Richardson DC, Richardson JS, Terwilliger TC, Zwart PH 2010 PHENIX: a comprehensive Python-based system for macromolecular structure solution. Acta Crystallogr D Biol Crystallogr 66:213–221. doi:10.1107/S0907444909052925. 20124702 PMC2815670

[103] Chen VB, Arendall WB III, Headd JJ, Keedy DA, Immormino RM, Kapral GJ, Murray LW, Richardson JS, Richardson DC 2010 MolProbity: all-atom structure validation for macromolecular crystallography. Acta Crystallogr D Biol Crystallogr 66:12–21. doi:10.1107/S0907444909042073. 20057044 PMC2803126

