# Supplementary material for: Interaction of the Ankyrin H Core Effector of Legionella with the Host LARP7 Component of the 7SK snRNP Complex
Source: mBio. 2019 Aug 27;10(4):e01942-19. doi: 10.1128/mBio.01942-19 (PMC6712400; doi:10.1128/mBio.01942-19)
Supplement: TABLE S3 [file mBio.01942-19-st003.docx]

**Nuclear Targeting of *Legionella* Core Effector AnkH and its Interaction with the Host LARP7**

**Supplemental Material**

**Table S3: Complete list of genes upregulated in hMDMs infected with Δ*ankH* null mutant compared to WT strain of *L. pneumophila*.**

| **ENSEMBL GENE** | **ENTREZ ID** | **GENE SYMBOL\|DESCRIPTION** | **log2FC**  **(ankh/wild_type)** | **p_value** |
| --- | --- | --- | --- | --- |
| ENSG00000120129 | 1843 | DUSP1\|dual specificity phosphatase 1 | 1.24951 | 5.00E-05 |
| ENSG00000120738 | 1958 | EGR1\|early growth response 1 | 2.25815 | 5.00E-05 |
| ENSG00000132002 | 3337 | DNAJB1\|DnaJ (Hsp40) homolog, subfamily B, member 1 | 1.51215 | 5.00E-05 |
| ENSG00000135549 | 5570 | PKIB\|protein kinase (cAMP-dependent, catalytic) inhibitor beta | 1.33129 | 0.00105 |
| ENSG00000151790 | 6999 | TDO2\|tryptophan 2,3-dioxygenase | 1.01074 | 0.0006 |
| ENSG00000152380 | 167555 | FAM151B\|family with sequence similarity 151, member B | 1.08992 | 0.00015 |
| ENSG00000164418 | 2898 | GRIK2\|glutamate receptor, ionotropic, kainate 2 | 1.78851 | 0.00245 |
| ENSG00000165694 | 90167 | FRMD7\|FERM domain containing 7 | 1.3049 | 5.00E-05 |
| ENSG00000170345 | 2353 | FOS\|FBJ murine osteosarcoma viral oncogene homolog | 1.15258 | 5.00E-05 |
| ENSG00000185842 | 127602 | DNAH14\|dynein, axonemal, heavy chain 14 | 1.00125 | 0.0014 |
| ENSG00000198300 | 5178 | PEG3\|paternally expressed 3 | 2.64637 | 0.0013 |
| ENSG00000204388 | 3304 | HSPA1B\|heat shock 70kDa protein 1B | 2.9979 | 5.00E-05 |
| ENSG00000214787 | 643680 | MS4A4E\|membrane-spanning 4-domains, subfamily A, member 4E | 2.36464 | 0.001 |
| ENSG00000225465 |  | RFPL1S\| | 2.61193 | 0.00195 |
| ENSG00000226047 |  |  | 1.04367 | 0.0009 |
| ENSG00000227028 | 100128590 | SLC8A1-AS1\|SLC8A1 antisense RNA 1 | 2.66729 | 5.00E-05 |
| ENSG00000229956 | 100852410 | ZRANB2-AS2\|ZRANB2 antisense RNA 2 (head to head) | 1.22918 | 0.00125 |
| ENSG00000234506 | 101927015 | LINC01506\|long intergenic non-protein coding RNA 1506 | 1.10281 | 5.00E-05 |
| ENSG00000245573 | 497258 | BDNF-AS\|BDNF antisense RNA | 1.01595 | 0.00595 |
| ENSG00000262097 | 101927311 | \|uncharacterized LOC101927311 | 1.32999 | 5.00E-05 |
| ENSG00000279348 |  |  | 1.02381 | 5.00E-05 |
|  |  |  |  |  |
