# Supplementary material for: Interaction of the Ankyrin H Core Effector of Legionella with the Host LARP7 Component of the 7SK snRNP Complex
Source: mBio. 2019 Aug 27;10(4):e01942-19. doi: 10.1128/mBio.01942-19 (PMC6712400; doi:10.1128/mBio.01942-19)
Supplement: TABLE S4 [file mBio.01942-19-st004.docx]

**Nuclear Targeting of *Legionella* Core Effector AnkH and its Interaction with the Host LARP7**

**Supplemental Material**

**Table S4: Complete list of genes downregulated in hMDMs infected with Δ*ankH* null mutant compared to WT strain of *L. pneumophila*.**

| **ENSEMBL GENE** | **ENTREZ ID** | **GENE SYMBOL\|DESCRIPTION** | **log2FC**  **(ankh/wild_type)** | **p_value** |
| --- | --- | --- | --- | --- |
| ENSG00000005075 | 5439 | POLR2J\|polymerase (RNA) II (DNA directed) polypeptide J, 13.3kDa | -1.00719 | 0.0004 |
| ENSG00000008382 | 84954 | MPND\|MPN domain containing | -1.13737 | 0.00305 |
| ENSG00000008441 | 4784 | NFIX\|nuclear factor I/X (CCAAT-binding transcription factor) | -1.56187 | 0.00215 |
| ENSG00000010295 | 25900 | IFFO1\|intermediate filament family orphan 1 | -1.02159 | 5.00E-05 |
| ENSG00000011028 | 9902 | MRC2\|mannose receptor, C type 2 | -1.58459 | 0.0001 |
| ENSG00000014164 | 23144 | ZC3H3\|zinc finger CCCH-type containing 3 | -1.16549 | 5.00E-05 |
| ENSG00000015285 | 7454 | WAS\|Wiskott-Aldrich syndrome | -1.02431 | 5.00E-05 |
| ENSG00000021762 | 114879 | OSBPL5\|oxysterol binding protein-like 5 | -1.0211 | 0.00325 |
| ENSG00000023191 | 6050 | RNH1\|ribonuclease/angiogenin inhibitor 1 | -1.21807 | 5.00E-05 |
| ENSG00000025770 | 29781 | NCAPH2\|non-SMC condensin II complex, subunit H2 | -1.01792 | 0.00085 |
| ENSG00000029534 | 286 | ANK1\|ankyrin 1, erythrocytic | -1.56839 | 0.00025 |
| ENSG00000030582 | 2896 | GRN\|granulin | -1.10623 | 5.00E-05 |
| ENSG00000037042 | 27175 | TUBG2\|tubulin, gamma 2 | -1.06627 | 0.001 |
| ENSG00000050820 | 9564 | BCAR1\|breast cancer anti-estrogen resistance 1 | -1.06863 | 0.00475 |
| ENSG00000051128 | 9454 | HOMER3\|homer scaffolding protein 3 | -1.92266 | 0.0026 |
| ENSG00000051523 | 1535 | CYBA\|cytochrome b-245, alpha polypeptide | -1.24454 | 5.00E-05 |
| ENSG00000061938 | 10188 | TNK2\|tyrosine kinase, non-receptor, 2 | -1.22665 | 0.00035 |
| ENSG00000063245 | 29924 | EPN1\|epsin 1 | -1.40878 | 5.00E-05 |
| ENSG00000063854 | 3029 | HAGH\|hydroxyacylglutathione hydrolase | -1.02578 | 0.003 |
| ENSG00000064490 | 8625 | RFXANK\|regulatory factor X-associated ankyrin-containing protein | -1.10454 | 0.00115 |
| ENSG00000064687 | 10347 | ABCA7\|ATP-binding cassette, sub-family A (ABC1), member 7 | -1.16013 | 0.00305 |
| ENSG00000065268 | 57418 | WDR18\|WD repeat domain 18 | -1.24648 | 0.0052 |
| ENSG00000065320 | 9423 | NTN1\|netrin 1 | -1.27328 | 0.00015 |
| ENSG00000068001 | 8692 | HYAL2\|hyaluronoglucosaminidase 2 | -1.02605 | 0.0037 |
| ENSG00000070404 | 10272 | FSTL3\|follistatin-like 3 (secreted glycoprotein) | -1.47082 | 0.00035 |
| ENSG00000070413 | 9993 | DGCR2\|DiGeorge syndrome critical region gene 2 | -1.08748 | 0.00105 |
| ENSG00000071859 | 9130 | FAM50A\|family with sequence similarity 50, member A | -1.03637 | 0.0007 |
| ENSG00000071889 | 60343 | FAM3A\|family with sequence similarity 3, member A | -1.10308 | 5.00E-05 |
| ENSG00000071894 | 29894 | CPSF1\|cleavage and polyadenylation specific factor 1, 160kDa | -1.89935 | 5.00E-05 |
| ENSG00000072110 | 87 | ACTN1\|actinin, alpha 1 | -1.02917 | 5.00E-05 |
| ENSG00000072786 | 6793 | STK10\|serine/threonine kinase 10 | -1.09231 | 5.00E-05 |
| ENSG00000074181 | 4854 | NOTCH3\|notch 3 | -1.57732 | 5.00E-05 |
| ENSG00000074964 | 55160 | ARHGEF10L\|Rho guanine nucleotide exchange factor (GEF) 10-like | -1.11483 | 0.00095 |
| ENSG00000075618 | 6624 | FSCN1\|fascin actin-bundling protein 1 | -1.54859 | 5.00E-05 |
| ENSG00000076924 | 56949 | XAB2\|XPA binding protein 2 | -1.45048 | 5.00E-05 |
| ENSG00000077454 | 4034 | LRCH4\|leucine-rich repeats and calponin homology (CH) domain containing 4 | -1.18425 | 5.00E-05 |
| ENSG00000078269 | 8871 | SYNJ2\|synaptojanin 2 | -1.03271 | 0.00515 |
| ENSG00000078808 | 51150 | SDF4\|stromal cell derived factor 4 | -1.34504 | 5.00E-05 |
| ENSG00000079432 | 23152 | CIC\|capicua transcriptional repressor | -1.26161 | 0.0007 |
| ENSG00000080573 | 50509 | COL5A3\|collagen, type V, alpha 3 | -1.26729 | 5.00E-05 |
| ENSG00000083838 | 55663 | ZNF446\|zinc finger protein 446 | -1.70362 | 0.00435 |
| ENSG00000085117 | 3732 | CD82\|CD82 molecule | -1.09771 | 5.00E-05 |
| ENSG00000088256 | 2767 | GNA11\|guanine nucleotide binding protein (G protein), alpha 11 (Gq class) | -1.47458 | 5.00E-05 |
| ENSG00000090013 | 645 | BLVRB\|biliverdin reductase B | -1.05725 | 5.00E-05 |
| ENSG00000095397 | 25861 | DFNB31\|deafness, autosomal recessive 31 | -1.94109 | 0.00035 |
| ENSG00000099817 | 5434 | POLR2E\|polymerase (RNA) II (DNA directed) polypeptide E, 25kDa | -1.17485 | 0.00135 |
| ENSG00000099821 | 5442 | POLRMT\|polymerase (RNA) mitochondrial (DNA directed) | -1.29291 | 0.00335 |
| ENSG00000099995 | 10291 | SF3A1\|splicing factor 3a, subunit 1, 120kDa | -1.15539 | 0.0006 |
| ENSG00000100056 | 8220 | DGCR14\|DiGeorge syndrome critical region gene 14 | -1.05868 | 0.00045 |
| ENSG00000100075 | 6576 | SLC25A1\|solute carrier family 25 (mitochondrial carrier; citrate transporter), member 1 | -1.07913 | 5.00E-05 |
| ENSG00000100097 | 3956 | LGALS1\|lectin, galactoside-binding, soluble, 1 | -1.05694 | 5.00E-05 |
| ENSG00000100147 | 79879 | CCDC134\|coiled-coil domain containing 134 | -1.1867 | 0.00015 |
| ENSG00000100241 | 6305 | SBF1\|SET binding factor 1 | -1.6413 | 0.00015 |
| ENSG00000100258 | 91289 | LMF2\|lipase maturation factor 2 | -1.59493 | 5.00E-05 |
| ENSG00000100292 | 3162 | HMOX1\|heme oxygenase 1 | -1.08059 | 5.00E-05 |
| ENSG00000100299 | 410 | ARSA\|arylsulfatase A | -1.05355 | 0.0006 |
| ENSG00000100300 | 706 | TSPO\|translocator protein (18kDa) | -1.12915 | 5.00E-05 |
| ENSG00000100319 | 55954 | ZMAT5\|zinc finger, matrin-type 5 | -1.14104 | 5.00E-05 |
| ENSG00000100417 | 5372 | PMM1\|phosphomannomutase 1 | -1.44872 | 5.00E-05 |
| ENSG00000100425 | 23774 | BRD1\|bromodomain containing 1 | -1.0918 | 0.0017 |
| ENSG00000100429 | 83933 | HDAC10\|histone deacetylase 10 | -1.30876 | 5.00E-05 |
| ENSG00000100949 | 5875 | RABGGTA\|Rab geranylgeranyltransferase, alpha subunit | -1.06608 | 5.00E-05 |
| ENSG00000100985 | 4318 | MMP9\|matrix metallopeptidase 9 | -1.06531 | 5.00E-05 |
| ENSG00000101194 | 63910 | SLC17A9\|solute carrier family 17 (vesicular nucleotide transporter), member 9 | -1.14327 | 5.00E-05 |
| ENSG00000101335 | 10398 | MYL9\|myosin, light chain 9, regulatory | -1.0549 | 0.00225 |
| ENSG00000101443 | 10406 | WFDC2\|WAP four-disulfide core domain 2 | -1.6624 | 0.0038 |
| ENSG00000101986 | 215 | ABCD1\|ATP-binding cassette, sub-family D (ALD), member 1 | -1.45046 | 5.00E-05 |
| ENSG00000101997 | 28952 | CCDC22\|coiled-coil domain containing 22 | -1.08562 | 5.00E-05 |
| ENSG00000102265 | 7076 | TIMP1\|TIMP metallopeptidase inhibitor 1 | -1.0606 | 5.00E-05 |
| ENSG00000102879 | 11151 | CORO1A\|coronin, actin binding protein, 1A | -1.06787 | 5.00E-05 |
| ENSG00000103145 | 54985 | HCFC1R1\|host cell factor C1 regulator 1 (XPO1 dependent) | -1.00296 | 0.00025 |
| ENSG00000103257 | 8140 | SLC7A5\|solute carrier family 7 (amino acid transporter light chain, L system), member 5 | -1.07431 | 5.00E-05 |
| ENSG00000103335 | 9780 | PIEZO1\|piezo-type mechanosensitive ion channel component 1 | -1.04014 | 0.003 |
| ENSG00000103653 | 1445 | CSK\|c-src tyrosine kinase | -1.20218 | 5.00E-05 |
| ENSG00000104368 | 5327 | PLAT\|plasminogen activator, tissue | -1.51427 | 5.00E-05 |
| ENSG00000104907 | 55621 | TRMT1\|tRNA methyltransferase 1 | -1.04471 | 0.00115 |
| ENSG00000104973 | 81857 | MED25\|mediator complex subunit 25 | -1.51656 | 0.0024 |
| ENSG00000104976 | 6618 | SNAPC2\|small nuclear RNA activating complex, polypeptide 2, 45kDa | -1.68231 | 0.00015 |
| ENSG00000105204 | 9149 | DYRK1B\|dual-specificity tyrosine-(Y)-phosphorylation regulated kinase 1B | -1.28731 | 0.0018 |
| ENSG00000105223 | 23646 | PLD3\|phospholipase D family, member 3 | -1.04403 | 5.00E-05 |
| ENSG00000105229 | 51588 | PIAS4\|protein inhibitor of activated STAT, 4 | -1.88599 | 0.00125 |
| ENSG00000105374 | 4818 | NKG7\|natural killer cell granule protein 7 | -1.11691 | 5.00E-05 |
| ENSG00000105669 | 11316 | COPE\|coatomer protein complex, subunit epsilon | -1.00067 | 0.00035 |
| ENSG00000105701 | 23770 | FKBP8\|FK506 binding protein 8, 38kDa | -1.50269 | 5.00E-05 |
| ENSG00000105717 | 80714 | PBX4\|pre-B-cell leukemia homeobox 4 | -1.63027 | 0.0019 |
| ENSG00000105723 | 2931 | GSK3A\|glycogen synthase kinase 3 alpha | -1.21067 | 5.00E-05 |
| ENSG00000105732 | 64763 | ZNF574\|zinc finger protein 574 | -1.53883 | 0.00015 |
| ENSG00000106009 | 221927 | BRAT1\|BRCA1-associated ATM activator 1 | -1.35966 | 0.00445 |
| ENSG00000106348 | 3614 | IMPDH1\|IMP (inosine 5'-monophosphate) dehydrogenase 1 | -1.2246 | 5.00E-05 |
| ENSG00000106683 | 3984 | LIMK1\|LIM domain kinase 1 | -1.28683 | 5.00E-05 |
| ENSG00000107816 | 84445 | LZTS2\|leucine zipper, putative tumor suppressor 2 | -1.12868 | 0.00105 |
| ENSG00000108639 | 9144 | SYNGR2\|synaptogyrin 2 | -1.0749 | 5.00E-05 |
| ENSG00000108840 | 10014 | HDAC5\|histone deacetylase 5 | -1.07427 | 0.00025 |
| ENSG00000109736 | 10227 | MFSD10\|major facilitator superfamily domain containing 10 | -1.23104 | 5.00E-05 |
| ENSG00000110025 | 29907 | SNX15\|sorting nexin 15 | -5.02403 | 0.0016 |
| ENSG00000110046 | 23130 | ATG2A\|autophagy related 2A | -1.21033 | 0.0002 |
| ENSG00000110446 | 51296 | SLC15A3\|solute carrier family 15 (oligopeptide transporter), member 3 | -1.04048 | 5.00E-05 |
| ENSG00000110717 | 4728 | NDUFS8\|NADH dehydrogenase (ubiquinone) Fe-S protein 8, 23kDa (NADH-coenzyme Q reductase) | -1.04348 | 5.00E-05 |
| ENSG00000110944 | 51561 | IL23A\|interleukin 23, alpha subunit p19 | -1.04283 | 5.00E-05 |
| ENSG00000111321 | 4055 | LTBR\|lymphotoxin beta receptor (TNFR superfamily, member 3) | -1.05988 | 5.00E-05 |
| ENSG00000111678 | 113246 | C12orf57\|chromosome 12 open reading frame 57 | -1.18795 | 0.0036 |
| ENSG00000113494 | 5618 | PRLR\|prolactin receptor | -1.28143 | 0.00215 |
| ENSG00000113657 | 1809 | DPYSL3\|dihydropyrimidinase-like 3 | -1.12821 | 5.00E-05 |
| ENSG00000114554 | 5361 | PLXNA1\|plexin A1 | -1.40661 | 0.00265 |
| ENSG00000114626 | 80325 | ABTB1\|ankyrin repeat and BTB (POZ) domain containing 1 | -1.08265 | 0.0018 |
| ENSG00000115085 | 7535 | ZAP70\|zeta-chain (TCR) associated protein kinase 70kDa | -2.2362 | 0.00025 |
| ENSG00000115286 | 374291 | NDUFS7\|NADH dehydrogenase (ubiquinone) Fe-S protein 7, 20kDa (NADH-coenzyme Q reductase) | -1.2702 | 0.00285 |
| ENSG00000115718 | 5624 | PROC\|protein C (inactivator of coagulation factors Va and VIIIa) | -1.5442 | 0.0027 |
| ENSG00000116691 | 60672 | MIIP\|migration and invasion inhibitory protein | -1.06346 | 5.00E-05 |
| ENSG00000116809 | 7709 | ZBTB17\|zinc finger and BTB domain containing 17 | -1.2056 | 5.00E-05 |
| ENSG00000117984 | 1509 | CTSD\|cathepsin D | -1.18663 | 5.00E-05 |
| ENSG00000120899 | 2185 | PTK2B\|protein tyrosine kinase 2 beta | -1.15075 | 5.00E-05 |
| ENSG00000120913 | 64236 | PDLIM2\|PDZ and LIM domain 2 (mystique) | -1.7752 | 5.00E-05 |
| ENSG00000120949 | 943 | TNFRSF8\|tumor necrosis factor receptor superfamily, member 8 | -1.03051 | 5.00E-05 |
| ENSG00000121057 | 8165 | AKAP1\|A kinase (PRKA) anchor protein 1 | -1.03472 | 0.00055 |
| ENSG00000123143 | 5585 | PKN1\|protein kinase N1 | -1.00546 | 5.00E-05 |
| ENSG00000123453 | 1757 | SARDH\|sarcosine dehydrogenase | -1.14408 | 0.0002 |
| ENSG00000124216 | 6615 | SNAI1\|snail family zinc finger 1 | -1.78349 | 0.00195 |
| ENSG00000125089 | 54436 | SH3TC1\|SH3 domain and tetratricopeptide repeats 1 | -1.0519 | 0.00015 |
| ENSG00000125148 | 4502 | MT2A\|metallothionein 2A | -1.06199 | 5.00E-05 |
| ENSG00000125503 | 54776 | PPP1R12C\|protein phosphatase 1, regulatory subunit 12C | -1.29062 | 5.00E-05 |
| ENSG00000125534 | 79144 | PPDPF\|pancreatic progenitor cell differentiation and proliferation factor | -1.42636 | 5.00E-05 |
| ENSG00000125656 | 8192 | CLPP\|caseinolytic mitochondrial matrix peptidase proteolytic subunit | -1.14732 | 5.00E-05 |
| ENSG00000125726 | 970 | CD70\|CD70 molecule | -1.07511 | 0.00035 |
| ENSG00000125817 | 1059 | CENPB\|centromere protein B, 80kDa | -1.06327 | 5.00E-05 |
| ENSG00000125912 | 56926 | NCLN\|nicalin | -1.60475 | 5.00E-05 |
| ENSG00000126062 | 11070 | TMEM115\|transmembrane protein 115 | -1.19645 | 5.00E-05 |
| ENSG00000126254 | 79171 | RBM42\|RNA binding motif protein 42 | -1.67206 | 5.00E-05 |
| ENSG00000126353 | 1236 | CCR7\|chemokine (C-C motif) receptor 7 | -1.03117 | 5.00E-05 |
| ENSG00000126461 | 58506 | SCAF1\|SR-related CTD-associated factor 1 | -1.67049 | 5.00E-05 |
| ENSG00000126561 | 6776 | STAT5A\|signal transducer and activator of transcription 5A | -1.08881 | 5.00E-05 |
| ENSG00000126903 | 8273 | SLC10A3\|solute carrier family 10, member 3 | -1.01445 | 5.00E-05 |
| ENSG00000126934 | 5605 | MAP2K2\|mitogen-activated protein kinase kinase 2 | -1.03897 | 5.00E-05 |
| ENSG00000127663 | 23030 | KDM4B\|lysine (K)-specific demethylase 4B | -1.5128 | 5.00E-05 |
| ENSG00000127666 | 148022 | TICAM1\|toll-like receptor adaptor molecule 1 | -1.0486 | 5.00E-05 |
| ENSG00000128228 | 23753 | SDF2L1\|stromal cell-derived factor 2-like 1 | -1.65596 | 5.00E-05 |
| ENSG00000128271 | 135 | ADORA2A\|adenosine A2a receptor | -1.51909 | 0.00415 |
| ENSG00000128342 | 3976 | LIF\|leukemia inhibitory factor | -1.17852 | 5.00E-05 |
| ENSG00000129911 | 83855 | KLF16\|Kruppel-like factor 16 | -1.04128 | 5.00E-05 |
| ENSG00000129925 | 58986 | TMEM8A\|transmembrane protein 8A | -1.35995 | 5.00E-05 |
| ENSG00000130165 | 84337 | ELOF1\|ELF1 homolog, elongation factor 1 | -1.04415 | 5.00E-05 |
| ENSG00000130203 | 348 | APOE\|apolipoprotein E | -1.35627 | 5.00E-05 |
| ENSG00000130222 | 10912 | GADD45G\|growth arrest and DNA-damage-inducible, gamma | -1.11982 | 0.0008 |
| ENSG00000130255 | 25873 | RPL36\|ribosomal protein L36 | -1.11055 | 0.0001 |
| ENSG00000130313 | 25796 | PGLS\|6-phosphogluconolactonase | -1.22736 | 5.00E-05 |
| ENSG00000130479 | 55201 | MAP1S\|microtubule-associated protein 1S | -1.1206 | 5.00E-05 |
| ENSG00000130706 | 11047 | ADRM1\|adhesion regulating molecule 1 | -1.1761 | 5.00E-05 |
| ENSG00000130726 | 10155 | TRIM28\|tripartite motif containing 28 | -1.0238 | 5.00E-05 |
| ENSG00000131165 | 5119 | CHMP1A\|charged multivesicular body protein 1A | -1.09835 | 5.00E-05 |
| ENSG00000131459 | 9945 | GFPT2\|glutamine-fructose-6-phosphate transaminase 2 | -1.10419 | 5.00E-05 |
| ENSG00000131653 | 84231 | TRAF7\|TNF receptor-associated factor 7, E3 ubiquitin protein ligase | -1.41604 | 0.00015 |
| ENSG00000131669 | 4814 | NINJ1\|ninjurin 1 | -1.35514 | 5.00E-05 |
| ENSG00000131759 | 5914 | RARA\|retinoic acid receptor, alpha | -1.14887 | 5.00E-05 |
| ENSG00000132017 | 90379 | DCAF15\|DDB1 and CUL4 associated factor 15 | -1.20722 | 0.00035 |
| ENSG00000132382 | 10514 | MYBBP1A\|MYB binding protein (P160) 1a | -1.00679 | 0.0008 |
| ENSG00000133027 | 10400 | PEMT\|phosphatidylethanolamine N-methyltransferase | -1.50047 | 5.00E-05 |
| ENSG00000133069 | 9911 | TMCC2\|transmembrane and coiled-coil domain family 2 | -1.31481 | 5.00E-05 |
| ENSG00000133275 | 1455 | CSNK1G2\|casein kinase 1, gamma 2 | -1.29917 | 0.00475 |
| ENSG00000135094 | 10993 | SDS\|serine dehydratase | -1.05989 | 5.00E-05 |
| ENSG00000135723 | 29109 | FHOD1\|formin homology 2 domain containing 1 | -1.22406 | 0.00095 |
| ENSG00000136286 | 64005 | MYO1G\|myosin IG | -1.06235 | 5.00E-05 |
| ENSG00000136717 | 274 | BIN1\|bridging integrator 1 | -1.00875 | 5.00E-05 |
| ENSG00000136877 | 2356 | FPGS\|folylpolyglutamate synthase | -1.20062 | 5.00E-05 |
| ENSG00000137166 | 116113 | FOXP4\|forkhead box P4 | -1.45906 | 0.0004 |
| ENSG00000137221 | 93643 | TJAP1\|tight junction associated protein 1 (peripheral) | -1.18844 | 0.00065 |
| ENSG00000137266 | 63027 | SLC22A23\|solute carrier family 22, member 23 | -1.00819 | 0.00505 |
| ENSG00000137818 | 6176 | RPLP1\|ribosomal protein, large, P1 | -1.18368 | 5.00E-05 |
| ENSG00000138080 | 11117 | EMILIN1\|elastin microfibril interfacer 1 | -1.52419 | 5.00E-05 |
| ENSG00000139567 | 94 | ACVRL1\|activin A receptor type II-like 1 | -1.09297 | 0.0025 |
| ENSG00000140548 | 374655 | ZNF710\|zinc finger protein 710 | -1.03225 | 0.0063 |
| ENSG00000140854 | 10300 | KATNB1\|katanin p80 (WD repeat containing) subunit B 1 | -1.00905 | 0.0025 |
| ENSG00000140939 | 8996 | NOL3\|nucleolar protein 3 (apoptosis repressor with CARD domain) | -1.33415 | 0.0001 |
| ENSG00000141526 | 9123 | SLC16A3\|solute carrier family 16 (monocarboxylate transporter), member 3 | -1.11006 | 0.00055 |
| ENSG00000141985 | 6455 | SH3GL1\|SH3-domain GRB2-like 1 | -1.01881 | 5.00E-05 |
| ENSG00000141994 | 56931 | DUS3L\|dihydrouridine synthase 3-like | -1.48655 | 0.00085 |
| ENSG00000142186 | 57410 | SCYL1\|SCY1-like, kinase-like 1 | -1.14476 | 5.00E-05 |
| ENSG00000142546 | 51070 | NOSIP\|nitric oxide synthase interacting protein | -1.00975 | 5.00E-05 |
| ENSG00000143373 | 57592 | ZNF687\|zinc finger protein 687 | -1.07338 | 0.0059 |
| ENSG00000143774 | 2987 | GUK1\|guanylate kinase 1 | -1.12173 | 5.00E-05 |
| ENSG00000144476 | 57007 | ACKR3\|atypical chemokine receptor 3 | -1.37842 | 0.0023 |
| ENSG00000144579 | 58190 | CTDSP1\|CTD (carboxy-terminal domain, RNA polymerase II, polypeptide A) small phosphatase 1 | -1.14305 | 5.00E-05 |
| ENSG00000145901 | 10318 | TNIP1\|TNFAIP3 interacting protein 1 | -1.22933 | 5.00E-05 |
| ENSG00000145936 | 3779 | KCNMB1\|potassium channel subfamily M regulatory beta subunit 1 | -1.08752 | 5.00E-05 |
| ENSG00000147443 | 9046 | DOK2\|docking protein 2, 56kDa | -1.19973 | 5.00E-05 |
| ENSG00000148343 | 84895 | FAM73B\|family with sequence similarity 73, member B | -1.53125 | 0.0006 |
| ENSG00000149781 | 83706 | FERMT3\|fermitin family member 3 | -1.03321 | 5.00E-05 |
| ENSG00000149782 | 5331 | PLCB3\|phospholipase C, beta 3 (phosphatidylinositol-specific) | -1.09659 | 5.00E-05 |
| ENSG00000149925 | 226 | ALDOA\|aldolase A, fructose-bisphosphate | -1.32281 | 5.00E-05 |
| ENSG00000150672 | 1740 | DLG2\|discs, large homolog 2 (Drosophila) | -1.14705 | 0.0054 |
| ENSG00000151651 | 101 | ADAM8\|ADAM metallopeptidase domain 8 | -1.08517 | 5.00E-05 |
| ENSG00000153443 | 124402 | UBALD1\|UBA-like domain containing 1 | -2.02675 | 0.00385 |
| ENSG00000154099 | 123872 | DNAAF1\|dynein, axonemal, assembly factor 1 | -1.18584 | 0.0001 |
| ENSG00000156966 | 93010 | B3GNT7\|UDP-GlcNAc:betaGal beta-1,3-N-acetylglucosaminyltransferase 7 | -1.07511 | 0.00015 |
| ENSG00000157353 | 197258 | FUK\|fucokinase | -1.20746 | 5.00E-05 |
| ENSG00000158517 | 653361 | NCF1\|neutrophil cytosolic factor 1 | -1.18042 | 5.00E-05 |
| ENSG00000158941 | 57805 | CCAR2\|cell cycle and apoptosis regulator 2 | -1.03328 | 0.0002 |
| ENSG00000159069 | 54461 | FBXW5\|F-box and WD repeat domain containing 5 | -1.37742 | 0.0044 |
| ENSG00000159166 | 3898 | LAD1\|ladinin 1 | -1.50991 | 0.00365 |
| ENSG00000159189 | 714 | C1QC\|complement component 1, q subcomponent, C chain | -1.17726 | 5.00E-05 |
| ENSG00000159314 | 201176 | ARHGAP27\|Rho GTPase activating protein 27 | -1.75118 | 0.0017 |
| ENSG00000159363 | 23400 | ATP13A2\|ATPase type 13A2 | -1.22485 | 0.00035 |
| ENSG00000159496 | 266747 | RGL4\|ral guanine nucleotide dissociation stimulator-like 4 | -2.44684 | 0.00025 |
| ENSG00000160211 | 2539 | G6PD\|glucose-6-phosphate dehydrogenase | -1.21866 | 5.00E-05 |
| ENSG00000160285 | 4047 | LSS\|lanosterol synthase (2,3-oxidosqualene-lanosterol cyclase) | -1.12374 | 5.00E-05 |
| ENSG00000160326 | 11182 | SLC2A6\|solute carrier family 2 (facilitated glucose transporter), member 6 | -1.12955 | 5.00E-05 |
| ENSG00000160404 | 27433 | TOR2A\|torsin family 2, member A | -1.62058 | 5.00E-05 |
| ENSG00000160446 | 84885 | ZDHHC12\|zinc finger, DHHC-type containing 12 | -1.41004 | 5.00E-05 |
| ENSG00000160703 | 79671 | NLRX1\|NLR family member X1 | -1.04198 | 0.0013 |
| ENSG00000160789 | 4000 | LMNA\|lamin A/C | -1.37469 | 5.00E-05 |
| ENSG00000160877 | 112939 | NACC1\|nucleus accumbens associated 1, BEN and BTB (POZ) domain containing | -1.09164 | 5.00E-05 |
| ENSG00000161011 | 8878 | SQSTM1\|sequestosome 1 | -1.15104 | 5.00E-05 |
| ENSG00000161016 | 6132 | RPL8\|ribosomal protein L8 | -1.01671 | 5.00E-05 |
| ENSG00000161653 | 162417 | NAGS\|N-acetylglutamate synthase | -1.02547 | 0.00445 |
| ENSG00000162104 | 115 | ADCY9\|adenylate cyclase 9 | -1.14224 | 0.0039 |
| ENSG00000162302 | 8986 | RPS6KA4\|ribosomal protein S6 kinase, 90kDa, polypeptide 4 | -1.01178 | 0.0002 |
| ENSG00000162729 | 93185 | IGSF8\|immunoglobulin superfamily, member 8 | -1.28496 | 5.00E-05 |
| ENSG00000162897 | 83953 | FCAMR\|Fc receptor, IgA, IgM, high affinity | -1.0373 | 5.00E-05 |
| ENSG00000163430 | 11167 | FSTL1\|follistatin-like 1 | -1.53001 | 0.00275 |
| ENSG00000163702 | 84818 | IL17RC\|interleukin 17 receptor C | -1.2268 | 5.00E-05 |
| ENSG00000163870 | 131601 | TPRA1\|transmembrane protein, adipocyte asscociated 1 | -1.11974 | 5.00E-05 |
| ENSG00000163931 | 7086 | TKT\|transketolase | -1.00824 | 5.00E-05 |
| ENSG00000164896 | 10922 | FASTK\|Fas-activated serine/threonine kinase | -1.07824 | 5.00E-05 |
| ENSG00000164897 | 83590 | TMUB1\|transmembrane and ubiquitin-like domain containing 1 | -1.22881 | 0.0008 |
| ENSG00000165178 | 654817 | NCF1C\|neutrophil cytosolic factor 1C pseudogene | -1.27805 | 5.00E-05 |
| ENSG00000165233 | 84270 | CARD19\|chromosome 9 open reading frame 89 | -1.44697 | 5.00E-05 |
| ENSG00000165886 | 80019 | UBTD1\|ubiquitin domain containing 1 | -1.08225 | 5.00E-05 |
| ENSG00000166133 | 27079 | RPUSD2\|RNA pseudouridylate synthase domain containing 2 | -1.01157 | 5.00E-05 |
| ENSG00000166140 | 84936 | ZFYVE19\|zinc finger, FYVE domain containing 19 | -1.02447 | 0.00435 |
| ENSG00000166165 | 1152 | CKB\|creatine kinase, brain | -2.00337 | 5.00E-05 |
| ENSG00000166189 | 79803 | HPS6\|Hermansky-Pudlak syndrome 6 | -1.07737 | 5.00E-05 |
| ENSG00000166484 | 5598 | MAPK7\|mitogen-activated protein kinase 7 | -1.02829 | 5.00E-05 |
| ENSG00000166816 | 197257 | LDHD\|lactate dehydrogenase D | -1.10143 | 0.00285 |
| ENSG00000166831 | 348093 | RBPMS2\|RNA binding protein with multiple splicing 2 | -1.04779 | 0.00085 |
| ENSG00000166925 | 81628 | TSC22D4\|TSC22 domain family, member 4 | -1.25566 | 5.00E-05 |
| ENSG00000167173 | 56905 | C15orf39\|chromosome 15 open reading frame 39 | -1.15298 | 0.0007 |
| ENSG00000167302 | 146705 | ENTHD2\|ENTH domain containing 2 | -1.79226 | 0.0001 |
| ENSG00000167508 | 4597 | MVD\|mevalonate (diphospho) decarboxylase | -1.50744 | 5.00E-05 |
| ENSG00000167578 | 53916 | RAB4B\|RAB4B, member RAS oncogene family | -1.0446 | 5.00E-05 |
| ENSG00000167657 | 1613 | DAPK3\|death-associated protein kinase 3 | -1.91505 | 5.00E-05 |
| ENSG00000167703 | 124935 | SLC43A2\|solute carrier family 43 (amino acid system L transporter), member 2 | -1.24864 | 5.00E-05 |
| ENSG00000167747 | 84798 | C19orf48\|chromosome 19 open reading frame 48 | -1.0195 | 0.00565 |
| ENSG00000167779 | 3489 | IGFBP6\|insulin-like growth factor binding protein 6 | -1.10574 | 5.00E-05 |
| ENSG00000167797 | 10263 | CDK2AP2\|cyclin-dependent kinase 2 associated protein 2 | -1.05986 | 5.00E-05 |
| ENSG00000167895 | 147138 | TMC8\|transmembrane channel-like 8 | -2.75204 | 5.00E-05 |
| ENSG00000167962 | 90850 | ZNF598\|zinc finger protein 598 | -1.11773 | 0.0013 |
| ENSG00000168056 | 4054 | LTBP3\|latent transforming growth factor beta binding protein 3 | -1.12471 | 0.0055 |
| ENSG00000168071 | 283234 | CCDC88B\|coiled-coil domain containing 88B | -1.58332 | 0.00395 |
| ENSG00000168096 | 124401 | ANKS3\|ankyrin repeat and sterile alpha motif domain containing 3 | -1.20393 | 0.00395 |
| ENSG00000168487 | 649 | BMP1\|bone morphogenetic protein 1 | -1.11063 | 0.0003 |
| ENSG00000168528 | 347735 | SERINC2\|serine incorporator 2 | -1.73007 | 5.00E-05 |
| ENSG00000169026 | 84179 | MFSD7\|major facilitator superfamily domain containing 7 | -1.08125 | 0.0011 |
| ENSG00000169188 | 27301 | APEX2\|APEX nuclease (apurinic/apyrimidinic endonuclease) 2 | -1.21942 | 5.00E-05 |
| ENSG00000169692 | 10555 | AGPAT2\|1-acylglycerol-3-phosphate O-acyltransferase 2 | -1.47395 | 5.00E-05 |
| ENSG00000169710 | 2194 | FASN\|fatty acid synthase | -1.67319 | 0.00155 |
| ENSG00000169738 | 51181 | DCXR\|dicarbonyl/L-xylulose reductase | -1.2648 | 5.00E-05 |
| ENSG00000169976 | 83443 | SF3B5\|splicing factor 3b, subunit 5, 10kDa | -1.05208 | 5.00E-05 |
| ENSG00000170458 | 929 | CD14\|CD14 molecule | -1.1104 | 0.0002 |
| ENSG00000170638 | 80305 | TRABD\|TraB domain containing | -1.48765 | 0.0003 |
| ENSG00000170906 | 4696 | NDUFA3\|NADH dehydrogenase (ubiquinone) 1 alpha subcomplex, 3, 9kDa | -1.03021 | 0.00455 |
| ENSG00000171105 | 3643 | INSR\|insulin receptor | -1.09733 | 0.002 |
| ENSG00000171298 | 2548 | GAA\|glucosidase, alpha; acid | -1.31969 | 5.00E-05 |
| ENSG00000172183 | 3669 | ISG20\|interferon stimulated exonuclease gene 20kDa | -1.3279 | 0.0035 |
| ENSG00000172354 | 2783 | GNB2\|guanine nucleotide binding protein (G protein), beta polypeptide 2 | -1.24667 | 5.00E-05 |
| ENSG00000172375 | 9854 | C2CD2L\|C2CD2-like | -1.04602 | 0.0033 |
| ENSG00000172543 | 1521 | CTSW\|cathepsin W | -1.00814 | 0.00015 |
| ENSG00000172663 | 80194 | TMEM134\|transmembrane protein 134 | -1.30309 | 5.00E-05 |
| ENSG00000172724 | 6363 | CCL19\|chemokine (C-C motif) ligand 19 | -1.52581 | 5.00E-05 |
| ENSG00000173264 | 56834 | GPR137\|G protein-coupled receptor 137 | -1.33788 | 5.00E-05 |
| ENSG00000173369 | 713 | C1QB\|complement component 1, q subcomponent, B chain | -1.0098 | 5.00E-05 |
| ENSG00000173372 | 712 | C1QA\|complement component 1, q subcomponent, A chain | -1.07418 | 5.00E-05 |
| ENSG00000173457 | 26472 | PPP1R14B\|protein phosphatase 1, regulatory (inhibitor) subunit 14B | -1.07777 | 5.00E-05 |
| ENSG00000173540 | 29925 | GMPPB\|GDP-mannose pyrophosphorylase B | -1.1117 | 0.00015 |
| ENSG00000173546 | 1464 | CSPG4\|chondroitin sulfate proteoglycan 4 | -1.1675 | 5.00E-05 |
| ENSG00000174775 | 3265 | HRAS\|Harvey rat sarcoma viral oncogene homolog | -1.59219 | 5.00E-05 |
| ENSG00000174886 | 126328 | NDUFA11\|NADH dehydrogenase (ubiquinone) 1 alpha subcomplex, 11, 14.7kDa | -1.18046 | 0.0001 |
| ENSG00000174938 | 26470 | SEZ6L2\|seizure related 6 homolog (mouse)-like 2 | -1.28382 | 5.00E-05 |
| ENSG00000174939 | 253982 | ASPHD1\|aspartate beta-hydroxylase domain containing 1 | -1.0931 | 0.00105 |
| ENSG00000175573 | 83638 | C11orf68\|chromosome 11 open reading frame 68 | -1.06967 | 5.00E-05 |
| ENSG00000175756 | 54998 | AURKAIP1\|aurora kinase A interacting protein 1 | -1.36108 | 5.00E-05 |
| ENSG00000176101 | 8636 | SSNA1\|Sjogren syndrome nuclear autoantigen 1 | -1.53953 | 5.00E-05 |
| ENSG00000176170 | 8877 | SPHK1\|sphingosine kinase 1 | -1.0981 | 0.0046 |
| ENSG00000176454 | 254531 | LPCAT4\|lysophosphatidylcholine acyltransferase 4 | -1.31541 | 0.0005 |
| ENSG00000176973 | 23625 | FAM89B\|family with sequence similarity 89, member B | -1.24525 | 5.00E-05 |
| ENSG00000176978 | 29952 | DPP7\|dipeptidyl-peptidase 7 | -1.53086 | 5.00E-05 |
| ENSG00000177030 | 10522 | DEAF1\|DEAF1 transcription factor | -1.01725 | 0.00495 |
| ENSG00000177106 | 64787 | EPS8L2\|EPS8-like 2 | -1.39058 | 0.0013 |
| ENSG00000177542 | 79751 | SLC25A22\|solute carrier family 25 (mitochondrial carrier: glutamate), member 22 | -1.57688 | 0.00015 |
| ENSG00000177600 | 6181 | RPLP2\|ribosomal protein, large, P2 | -1.01189 | 5.00E-05 |
| ENSG00000178209 | 5339 | PLEC\|plectin | -1.41348 | 5.00E-05 |
| ENSG00000178605 | 8225 | GTPBP6\|GTP binding protein 6 (putative) | -1.08428 | 5.00E-05 |
| ENSG00000179409 | 50628 | GEMIN4\|gem (nuclear organelle) associated protein 4 | -1.17819 | 5.00E-05 |
| ENSG00000179593 | 247 | ALOX15B\|arachidonate 15-lipoxygenase, type B | -1.01864 | 5.00E-05 |
| ENSG00000179922 | 147808 | ZNF784\|zinc finger protein 784 | -1.28387 | 0.00235 |
| ENSG00000180448 | 23526 | HMHA1\|histocompatibility (minor) HA-1 | -1.99385 | 5.00E-05 |
| ENSG00000181577 | 221416 | C6orf223\|chromosome 6 open reading frame 223 | -1.01552 | 0.00015 |
| ENSG00000182087 | 91304 | TMEM259\|transmembrane protein 259 | -1.03373 | 5.00E-05 |
| ENSG00000182487 | 654816 | NCF1B\|neutrophil cytosolic factor 1B pseudogene | -1.20503 | 5.00E-05 |
| ENSG00000182504 | 79598 | CEP97\|centrosomal protein 97kDa | -2.16027 | 5.00E-05 |
| ENSG00000183092 | 57596 | BEGAIN\|brain-enriched guanylate kinase-associated | -2.03118 | 0.00305 |
| ENSG00000183570 | 54039 | PCBP3\|poly(rC) binding protein 3 | -1.32106 | 0.0008 |
| ENSG00000183684 | 10189 | ALYREF\|Aly/REF export factor | -1.21359 | 0.0002 |
| ENSG00000183751 | 10607 | TBL3\|transducin (beta)-like 3 | -1.49669 | 5.00E-05 |
| ENSG00000184281 | 10078 | TSSC4\|tumor suppressing subtransferable candidate 4 | -1.44619 | 0.00075 |
| ENSG00000184489 | 11156 | PTP4A3\|protein tyrosine phosphatase type IVA, member 3 | -1.09171 | 0.00025 |
| ENSG00000184730 | 55911 | APOBR\|apolipoprotein B receptor | -1.1535 | 5.00E-05 |
| ENSG00000184897 | 8971 | H1FX\|H1 histone family, member X | -1.03817 | 0.0001 |
| ENSG00000185033 | 10509 | SEMA4B\|sema domain, immunoglobulin domain (Ig), transmembrane domain (TM) and short cytoplasmic domain, (semaphorin) 4B | -1.18097 | 5.00E-05 |
| ENSG00000185187 | 59307 | SIGIRR\|single immunoglobulin and toll-interleukin 1 receptor (TIR) domain | -1.21446 | 0.0011 |
| ENSG00000185201 | 10581 | IFITM2\|interferon induced transmembrane protein 2 | -1.04698 | 5.00E-05 |
| ENSG00000185504 | 80233 | FAAP100\|Fanconi anemia core complex associated protein 100 | -1.02877 | 0.00285 |
| ENSG00000185507 | 3665 | IRF7\|interferon regulatory factor 7 | -1.52581 | 0.00025 |
| ENSG00000185669 | 333929 | SNAI3\|snail family zinc finger 3 | -1.06225 | 0.0014 |
| ENSG00000185803 | 79581 | SLC52A2\|solute carrier family 52 (riboflavin transporter), member 2 | -1.02906 | 0.00315 |
| ENSG00000185885 | 8519 | IFITM1\|interferon induced transmembrane protein 1 | -1.3133 | 0.00055 |
| ENSG00000186010 | 51079 | NDUFA13\|NADH dehydrogenase (ubiquinone) 1 alpha subcomplex, 13 | -1.0379 | 5.00E-05 |
| ENSG00000186174 | 283149 | BCL9L\|B-cell CLL/lymphoma 9-like | -1.37879 | 0.0001 |
| ENSG00000186501 | 84065 | TMEM222\|transmembrane protein 222 | -1.30675 | 5.00E-05 |
| ENSG00000186635 | 116985 | ARAP1\|ArfGAP with RhoGAP domain, ankyrin repeat and PH domain 1 | -1.10892 | 5.00E-05 |
| ENSG00000186891 | 8784 | TNFRSF18\|tumor necrosis factor receptor superfamily, member 18 | -1.226 | 5.00E-05 |
| ENSG00000186907 | 349667 | RTN4RL2\|reticulon 4 receptor-like 2 | -1.7492 | 0.00185 |
| ENSG00000187531 | 51547 | SIRT7\|sirtuin 7 | -1.1814 | 0.00115 |
| ENSG00000187608 | 9636 | ISG15\|ISG15 ubiquitin-like modifier | -1.68484 | 0.00015 |
| ENSG00000187688 | 51393 | TRPV2\|transient receptor potential cation channel, subfamily V, member 2 | -1.06984 | 5.00E-05 |
| ENSG00000187838 | 57048 | TMEM256-PLSCR3\|phospholipid scramblase 3 | -1.15105 | 0.00045 |
| ENSG00000188372 | 7784 | ZP3\|zona pellucida glycoprotein 3 (sperm receptor) | -1.13303 | 5.00E-05 |
| ENSG00000188566 | 27158 | NDOR1\|NADPH dependent diflavin oxidoreductase 1 | -1.12096 | 0.0023 |
| ENSG00000188976 | 26155 | NOC2L\|NOC2-like nucleolar associated transcriptional repressor | -1.05362 | 5.00E-05 |
| ENSG00000189077 | 83862 | TMEM120A\|transmembrane protein 120A | -1.08651 | 5.00E-05 |
| ENSG00000196365 | 9361 | LONP1\|lon peptidase 1, mitochondrial | -1.0442 | 5.00E-05 |
| ENSG00000196453 | 27153 | ZNF777\|zinc finger protein 777 | -1.06024 | 5.00E-05 |
| ENSG00000196498 | 9612 | NCOR2\|nuclear receptor corepressor 2 | -1.03301 | 0.00045 |
| ENSG00000196576 | 23654 | PLXNB2\|plexin B2 | -1.37984 | 0.00055 |
| ENSG00000196843 | 10865 | ARID5A\|AT rich interactive domain 5A (MRF1-like) | -1.11183 | 5.00E-05 |
| ENSG00000196878 | 3914 | LAMB3\|laminin, beta 3 | -1.08183 | 0.0001 |
| ENSG00000196924 | 2316 | FLNA\|filamin A, alpha | -1.22521 | 5.00E-05 |
| ENSG00000197114 | 84619 | ZGPAT\|zinc finger, CCCH-type with G patch domain | -2.04932 | 0.00035 |
| ENSG00000197150 | 11194 | ABCB8\|ATP-binding cassette, sub-family B (MDR/TAP), member 8 | -1.24739 | 5.00E-05 |
| ENSG00000197272 | 246778 | IL27\|interleukin 27 | -1.55472 | 5.00E-05 |
| ENSG00000197903 | 85236 | HIST1H2BK\|histone cluster 1, H2bk | -1.34856 | 5.00E-05 |
| ENSG00000198026 | 63925 | ZNF335\|zinc finger protein 335 | -1.1129 | 5.00E-05 |
| ENSG00000198055 | 2870 | GRK6\|G protein-coupled receptor kinase 6 | -1.15645 | 5.00E-05 |
| ENSG00000198517 | 7975 | MAFK\|v-maf avian musculoaponeurotic fibrosarcoma oncogene homolog K | -1.40139 | 5.00E-05 |
| ENSG00000198917 | 51490 | C9orf114\|chromosome 9 open reading frame 114 | -1.48285 | 0.001 |
| ENSG00000205414 |  |  | -1.02484 | 0.00085 |
| ENSG00000211649 |  | IGLV7-46\| | -16.0296 | 0.0001 |
| ENSG00000211893 |  | IGHG2\| | -1.60157 | 0.0032 |
| ENSG00000211899 |  | IGHM\| | -1.09468 | 5.00E-05 |
| ENSG00000213145 | 1396 | CRIP1\|cysteine-rich protein 1 (intestinal) | -1.586 | 5.00E-05 |
| ENSG00000213689 | 11277 | TREX1\|three prime repair exonuclease 1 | -1.27944 | 0.00345 |
| ENSG00000213853 | 2013 | EMP2\|epithelial membrane protein 2 | -1.63523 | 0.00085 |
| ENSG00000213923 | 1454///102800317 | CSNK1E\|casein kinase 1, epsilon///CSNK1E\|LOC400927-CSNK1E readthrough | -1.18937 | 0.00225 |
| ENSG00000214063 | 7106 | TSPAN4\|tetraspanin 4 | -1.36378 | 0.00045 |
| ENSG00000218537 |  | MIF-AS1\| | -1.59613 | 0.0005 |
| ENSG00000221968 | 3995 | FADS3\|fatty acid desaturase 3 | -1.51122 | 5.00E-05 |
| ENSG00000225783 | 440823 | MIAT\|myocardial infarction associated transcript (non-protein coding) | -1.24161 | 0.00425 |
| ENSG00000226332 |  |  | -1.49849 | 0.00195 |
| ENSG00000228300 | 55009 | C19orf24\|chromosome 19 open reading frame 24 | -2.10381 | 0.0063 |
| ENSG00000230943 | 101927686 | \|uncharacterized LOC101927686 | -1.06553 | 5.00E-05 |
| ENSG00000235173 | 51236 | HGH1\|HGH1 homolog | -1.15194 | 0.001 |
| ENSG00000237989 | 101928399 | \|uncharacterized LOC101928399 | -1.12929 | 0.00015 |
| ENSG00000238227 | 90120 | C9orf69\|chromosome 9 open reading frame 69 | -1.00007 | 5.00E-05 |
| ENSG00000239857 | 51608 | GET4\|golgi to ER traffic protein 4 | -1.1015 | 0.00445 |
| ENSG00000241945 | 5822///102724159 | PWP2\|PWP2 periodic tryptophan protein homolog (yeast)///PWP2\|periodic tryptophan protein 2 homolog | -1.74723 | 0.0009 |
| ENSG00000242802 | 9907 | AP5Z1\|adaptor-related protein complex 5, zeta 1 subunit | -1.48415 | 5.00E-05 |
| ENSG00000243156 | 57553 | MICAL3\|microtubule associated monooxygenase, calponin and LIM domain containing 3 | -1.00651 | 0.0045 |
| ENSG00000249780 |  |  | -1.07823 | 0.0002 |
| ENSG00000254452 |  |  | -1.35845 | 0.0001 |
| ENSG00000254559 |  |  | -1.1664 | 0.0026 |
| ENSG00000254986 | 10072 | DPP3\|dipeptidyl-peptidase 3 | -1.10415 | 0.00015 |
| ENSG00000256007 |  | ARAP1-AS1\| | -1.71348 | 0.004 |
| ENSG00000257156 |  |  | -1.13237 | 0.0029 |
| ENSG00000257663 |  |  | -1.03551 | 0.0001 |
| ENSG00000261236 | 23246 | BOP1\|block of proliferation 1 | -1.50606 | 0.0024 |
| ENSG00000261796 | 100534599 | ISY1-RAB43\|ISY1-RAB43 readthrough | -1.10893 | 0.00035 |
| ENSG00000262049 |  |  | -1.24797 | 0.00605 |
| ENSG00000262413 |  |  | -1.08612 | 0.0005 |
| ENSG00000267436 |  |  | -1.09667 | 0.00075 |
| ENSG00000267519 | 284454 | \|uncharacterized LOC284454 | -1.17642 | 0.0003 |
| ENSG00000269858 | 112398 | EGLN2\|egl-9 family hypoxia-inducible factor 2 | -1.00968 | 5.00E-05 |
| ENSG00000269968 |  |  | -1.1508 | 0.00025 |
| ENSG00000272916 |  |  | -1.14253 | 0.0008 |
| ENSG00000273812 |  |  | -1.43518 | 0.00015 |
| ENSG00000275074 | 79873 | NUDT18\|nudix (nucleoside diphosphate linked moiety X)-type motif 18 | -1.35046 | 5.00E-05 |
| ENSG00000275294 |  |  | -1.08204 | 5.00E-05 |
