# Supplementary material for: Interaction of the Ankyrin H Core Effector of Legionella with the Host LARP7 Component of the 7SK snRNP Complex
Source: mBio. 2019 Aug 27;10(4):e01942-19. doi: 10.1128/mBio.01942-19 (PMC6712400; doi:10.1128/mBio.01942-19)
Supplement: TABLE S1 [file mBio.01942-19-st001.docx]

**Nuclear Targeting of *Legionella* Core Effector AnkH and its Interaction with the Host LARP7**

**Supplemental Material**

**Table S1. Primers used in this study.**

| ankH^E30T^ | F | CATATGGTTTTACTCCCCTCATAG |
| --- | --- | --- |
| ankH^E30T^ | R | TATCGATATCATCCAAAGATTCCC |
| ankH^Y31S^ | F | CTGGTTTTACTCCCCTCATAGAGT |
| ankH^Y31S^ | R | ATTCATCGATATCATCCAA |
| ankH^F33A^ | F | CTACTCCCCTCATAGAGTGTGCCA |
| ankH^F33A^ | R | CACCATATTCATCGATATCA |
| ankH^V63Y^ | F | ACACAGGACGCACTCCATTACATT |
| ankH^V63Y^ | R | AGTCTGGCTTGTTGATATCCACTT |
| ankH^T64E^ | F | AAGGACGCACTCCATTACATTGGG |
| ankH^T64E^ | R | CGACGTCTGGCTTGTTGATA |
| ankH^R96A^ | F | CTAATGGTCTTTGTGTATTGGTTT |
| ankH^R96A^ | R | CAGTGTAGGCATTAGGATCAGCGC |
| ankH^N97V^ | F | TTGGTCTTTGTGTATTGGTTTATC |
| ankH^N97V^ | R | CACGAGTGTAGGCATTAGGA |
| ankH^N59A^ | F | CAAGCCAGACGTCACAGGACGC |
| ankH^N59A^ | R | CGATATCCACTTTTCGAGCAATTAA |
| ankH^N92A^ | F | TTGCCTACACTCGTAATGGTCTT |
| ankH^N59A^ | R | CAGGATCAGCGCCGTAGGTTAA |
| ankH^H243D^ | F | AATGCCTTATGCTTTGTC |
| ankH^H243D^ | R | GCCACGACTCGCCGCAGG |
| ankH^D258A^ | F | CCAGGGGTGAAAATAGCTTACAAG |
| ankH^H243D^ | R | CAATTTTTGCCCACCACTGGTGAT |
| ankH^C324S^ | F | AGTTCGTGGGCTAATGTG |
| ankH^C324S^ | R | ATTTCCACTAATTTGAGA |

*All primers are 5’-phosphorylated. Orientation: F, forward; R, reverse
