# Supplementary material for: Interaction of the Ankyrin H Core Effector of Legionella with the Host LARP7 Component of the 7SK snRNP Complex
Source: mBio. 2019 Aug 27;10(4):e01942-19. doi: 10.1128/mBio.01942-19 (PMC6712400; doi:10.1128/mBio.01942-19)
Supplement: TABLE S2 [file mBio.01942-19-st002.docx]

**Nuclear Targeting of *Legionella* Core Effector AnkH and its Interaction with the Host LARP7**

**Supplemental Material**

**Table S2: Sample and Barcode Information**

| **No.** | **Sample-BMDM** | **Barcodes Used** |
| --- | --- | --- |
| 1 | Control_1 | 2 |
| 2 | Control_2 | 4 |
| 3 | Control_3 | 5 |
| 4 | Wildtype_1 | 6 |
| 5 | Wildtype_2 | 7 |
| 6 | Wildtype_3 | 12 |
| 10 | ankH_1 | 16 |
| 11 | ankH_2 | 18 |
| 12 | ankH_3 | 19 |
