# Supplementary material for: Interaction of the Ankyrin H Core Effector of Legionella with the Host LARP7 Component of the 7SK snRNP Complex
Source: mBio. 2019 Aug 27;10(4):e01942-19. doi: 10.1128/mBio.01942-19 (PMC6712400; doi:10.1128/mBio.01942-19)
Supplement: TABLE S5 [file mBio.01942-19-st005.docx]

**Nuclear Targeting of *Legionella* Core Effector AnkH and its Interaction with the Host LARP7**

**Supplemental Material**

**Table S5. Data collection and refinement**

|  | **SeMet AnkH** | **Native AnkH** |
| --- | --- | --- |
| **Data collection statistics** | | |
| Space group | P6_5_ 2 2 | P6_5_ 2 2 |
| *a,b,c* (Å), γ (º) | 100.3, 100.3, 266.6, 120 | 102.1, 102.1, 266.0, 120 |
| Wavelength (Å) | 0.9788 | 0.9795 |
| Resolution (Å) | 50-2.9 (2.95-2.90) | 51.1-2.45 (2.49-2.45) |
| Total Reflections | 930045 | 643977 |
| Unique reflections | 18541 | 30933 |
| R_meas_ | 0.117 (0.882) | 0.082 (0.855) |
| Completeness (%) | 96.8 (94.7) | 97.3 (94.4) |
| Redundancy | 50.2 (49.8) | 20.8 (21.3) |
| I/σ(*I*) | 49.3 (6.0) | 48.1 (6.1) |
| Wilson B (Å^2^) | 47.0 | 32.4 |
| **Refinement statistics** | | |
| R_cryst_ ^d^ / R_free_ ^e^ (%) |  | 0.172 / 0.210 |
| Rmsd on bonds (Å) |  | 0.004 |
| Rmsd on angles (º) |  | 0.601 |
| Favored (%) |  | 98.25 |
| Allowed (%) |  | 1.75 |
| PDB code |  | 6MCA |
